# Serum lipidomics‐based study of electroacupuncture for skin wound repair in rats

**DOI:** 10.1111/jcmm.17891

**Published:** 2023-07-30

**Authors:** Weibin Du, Lihong He, Zhenwei Wang, Yi Dong, Xiaofen He, Jintao Hu, Min Zhang

**Affiliations:** ^1^ Research Institute of Orthopaedics The Affiliated Jiangnan Hospital of Zhejiang Chinese Medical University Zhejiang China; ^2^ Hangzhou Xiaoshan Hospital of Traditional Chinese Medicine Zhejiang China; ^3^ Shaoxing TCM Hospital Affiliated to Zhejiang Chinese Medical University Zhejiang China; ^4^ Key Laboratory of Acupuncture and Neurology of Zhejiang Province, Department of Neurobiology and Acupuncture Research Zhejiang Chinese Medical University, The Third Clinical Medical College Zhejiang China; ^5^ Orthopaedics and Traumatology Department Hangzhou TCM Hospital Affiliated to Zhejiang Chinese Medical University Zhejiang China

**Keywords:** blood perfusion, electroacupuncture, lipid metabolomics, serum, wound repair

## Abstract

Lipid metabolism plays an important role in the repair of skin wounds. Studies have shown that acupuncture is very effective in skin wound repair. However, there is little knowledge about the mechanism of electroacupuncture. Thirty‐six SD rats were divided into three groups: sham‐operated group, model group and electroacupuncture group, with six rats in each group. After the intervention, orbital venous blood was collected for lipid metabolomics analysis, wound perfusion was detected and finally the effect of electroacupuncture on skin wound repair was comprehensively evaluated by combining wound healing rate and histology. Lipid metabolomics analysis revealed 11 differential metabolites in the model versus sham‐operated group. There were 115 differential metabolites in the model versus electro‐acupuncture group. 117 differential metabolites in the electro‐acupuncture versus sham‐operated group. There were two differential metabolites common to all three groups. Mainly cholesteryl esters and sphingolipids were elevated after electroacupuncture and triglycerides were largely decreased after electroacupuncture. The electroacupuncture group recovered faster than the model group in terms of blood perfusion and wound healing (p < 0.05). Electroacupuncture may promote rat skin wound repair by improving lipid metabolism and improving local perfusion.

## 1 INTRODUCTION

The skin is the largest organ of the body and is the first line of defence against external invasion and has properties that protect against microbial, mechanical, chemical, osmotic and thermal damage.^1^, ^2^ Skin trauma is very common in surgery and is characterized by high morbidity and complications which adversely affect the quality of life of patients and cause high global health system costs.^3^, ^4^ As patients' expectations of the speed and quality of wound healing increase, there is a need to seek additional treatment strategies that can promote rapid and high‐quality healing of skin wounds.

Acupuncture is an important part of Chinese traditional medicine, and plays an important role in clinical treatment. Acupuncture can promote wound healing through anti‐inflammatory effects improving microcirculation, and increasing re‐epithelialization and angiogenesis.^5^, ^6^ Electrical stimulation is widely used as a physical stimulus in the medical field and is gaining more and more attention in the area of skin wound repair.^7^, ^8^ Electrical stimulation accelerates wound healing by promoting cell proliferation and migration and regulating the expression of growth factors and is a green therapy that mobilizes endogenous currents in the skin and corrects the internal environment of the wounds.^9^ Electroacupuncture, on the other hand, embodies the combination of traditional Chinese medicine and modern medicine, combining acupuncture with electrical stimulation to bring out its greater benefits. Electroacupuncture for wound repair is safe, non‐invasive, effective and easy to use. However, the role and mechanism of electroacupuncture for cutaneous wound healing are less reported and need further study.

Skin wound healing involves several processes, including extracellular matrix remodelling, synthesis of pro‐inflammatory mediators and angiogenesis. The new blood vessels formed by repair can promote tissue blood perfusion and is a key factor in determining the quality of skin wound healing. Therefore, blood perfusion is essential in skin wound healing.^10^, ^11^ Human skin homeostasis requires the regulation of lipid metabolic balance and lipids secreted by sebocytes together with lipids derived from keratin‐forming cells form an important component of the skin barrier.^12^ Chromatography‐mass spectrometry (LC–MS) combines the capabilities of both HPLC and MS, significantly improving the analytical platform and providing a sensitive and specific tool for the identification and quantification of lipids.^13^, ^14^ However, there are fewer reports on the mechanism of whether electroac upuncture can promote skin wound healing by regulating serum lipid metabolism. In order to better understand the mechanism of skin wound healing by electroacupuncture, we established a rat sk in model of total skin defect. This study focused on the changes in serum lipid metabolites, related lipid pathways and wound blood perfusion during electroacupuncture intervention in rats with total skin defects to promote skin wound repair.

## 2 METHOD

### 2.1 Experimental animals

Eighteen male SD rats (weight 160 ± 20 g) were grouped in the Animal Experimentation Centre of Zhejiang University of Traditional Chinese Medicine for 1 week after acclimatization. SD rats were purchased and fed, and other animal procedures followed the animal research guidelines of the National Institutes of Health and the Animal Research Committee. And approved by the Experimental Animal Ethics Committee of Zhejiang University of Traditional Chinese Medicine (NO. IACUC‐20220221‐19).

### 2.2 Model preparation

Combined with the group's previous modelling basis, a full‐layer skin defect model was prepared. After anaesthesia, the modelling area (2 cm on the left and right side of the spine) was fixed at five points, trimmed and dehaired, rinsed with saline, disinfected with iodophor and deiodinated with ethanol. A 1*1 cm square model of the full skin defect was created using surgical scissors. Post‐operatively, the wound was naturally haemostatic and the wound was kept dry to prevent wound infection.

### 2.3 Grouping and processing

Eighteen SD rats were randomly divided into three groups of six rats each according to the table of random number methods, as follows: sham‐operated group, model group and electroacupuncture group. (1) Sham‐operated group: only hair clipping was done in the modelling area, and no wound model was made. (2) Model group: full skin defect model was prepared, and only iodophor was given daily for routine disinfection to prevent wound infection. (3) Electroacupuncture group: Based on the operation of the model group, electroacupuncture treatment was started on the same day. The central point of the wound and the normal skin at the edge of the wound (the edge of the midpoint of the four sides of the square wound) were selected and given electroacupuncture stimulation. The negative electrode was located at the centre and the positive electrode was located at the edge of the midpoint of the four sides of the square wound. Continuous pulses with a frequency of 2 Hz and an output current of 0.3 mA was used to stimulate each point successively for 5 min at each point, once a day for a total time of 20 min to prevent wound infection.

### 2.4 LC–MS model preparation

Orbital venous blood was taken from each group after 7 days. Take 200 μL of serum into a 1.5 mL centrifuge tube, add 800 μL of isopropanol and vortex for 1 min. After 30 min of ultrasonication in an ice bath, the samples were vortexed for 1 min and placed in a refrigerator at −20°C overnight. The next day, the centrifuge was pre‐cooled to 4°C and centrifuged at 13000 rpm for 15 min. 600 μL of Serum supernatant was taken, blown to dryness with nitrogen and 150 μL of isopropanol was re‐dissolved and centrifuged at 13000 rpm for 15 min. 100 μL of supernatant was taken into the injection vial for sample injection. The supernatant of the homogenate was centrifuged in equal amounts, blown dry with nitrogen and re‐dissolved to prepare QC samples. The liquid conditions were set and the samples were analysed by UHPLC‐QTOF/MS.

### 2.5 UHPLC‐QTOF/MS analysis

#### 2.5.1 Chromatography

Chromatographic separation was performed on an ExionLC system (AB Sciex). A Waters Acquity HSS T3 column (2.1 × 100 mm, 1.7 μm) was applied at the temperature of 55°C. Mobile phase A: water‐acetonitrile (40:60, containing 0.1% formic acid with 5 mM ammonium formate); mobile phase B: isopropanol‐acetonitrile (90:10, containing 0.1% formic acid with 5 mM ammonium formate) The gradient was optimized as follows: 0–13 min from 30% to 80% B, 13–20 min from 80% to 90% B, 20–21 min from 90% to 100% B, 21–26 min at 100% B, then back to the initial ratio of 30% B and maintained with additional 8 min for re‐equilibration. The injection volume of all samples was 2 μL.

#### 2.5.2 Mass spectrometry

To provide high‐resolution detection, an X500B Q‐TOF mass spectrometer (AB Sciex) equipped with an electrospray ionization source (Turbo Ionspray) was applied. MS detection was implemented both in negative and positive ion mode with the mass rang at m/z 150–1050. The parameters of the mass spectrometer were summarized as follows: gas1 and gas2, 45 psi; curtain gas, 35 psi. Heat block temperature, 550°C; ion spray voltage, − 4.5 kV in negative mode and 5.5 kV in positive; declustering potential, 50 V; collision energy, ±35 V; and the collision energy spread (CES) was ±15 V. To monitor the reproducibility and stability of the acquisition system, QC samples were prepared by pooling small aliquots of each sample. The QC specimens were analysed every six samples throughout the whole analysis procedure.

#### 2.5.3 Data processing

The raw profiles were extracted by SCIEX OS Analytics and transformed into a data matrix, mainly including information on the mass‐to‐charge ratio (m/z) and retention time (Rt) and peak area (intensity). All data were normalized by the total peak area and the Excel sheet was generated for subsequent metabolome analysis. To reduce signal interference from chance errors, variables with RSD ≥40% in QC were excluded in excel first.

The Excel files were imported into SIMCA 14.1 (Umetrics) software for multivariate mathematical and statistical analysis. Principal component analysis (PCA) was used to observe the overall distribution of the samples. In addition, the consistency of the samples within the group was analysed by PCA‐Class analysis. Generally, when a sample falls outside the ‘2‐std. dev.’ line under a principal component, the sample was considered abnormal data and the sample data was considered to be excluded before the subsequent analysis.

The OPLS‐DA replacement test statistically analyzes the validity of the OPLS‐DA model when Q2 intersects with the y‐axis at a negative value, indicating that the model is valid, which in turn screens for differential metabolites. Based on this model, the differential variables were screened according to the variable projection importance index (VIP value), and the variables with VIP >1 were generally considered to be meaningful variables causing the differences. The variables with a high impact on OPLS‐DA model building were further screened by the partial correlation coefficient (pcorr). Finally, the screened variables were tested for significance (Mann–Whitney Test), and a *p* < 0.05 was considered a significant difference variable. Potential markers were identified by HMDB (http://www.hmdb.ca/) and LIPID MAPS (https://www.lipidmaps.org/). All differential metabolites involved in the three groups were summarized by Venn diagrams. Heat maps of the differential metabolites were produced to visualize the high and low levels of the response of the compounds after the intervention, and corresponding bar charts were produced for further analysis. Based on the results of the significantly different metabolites screened and identified, the compound name results for each group were imported into Metabo Analyst 5.0 (http://www.MetaboAnalyst.ca/) for metabolic pathway analysis.

### 2.6 Laser doppler perfusion imaging test

The groups were examined at 1d, 4d and 7d using laser Doppler perfusion imaging with a distance of 10 cm between the probe and the test object and an imaging range of 1.0 cm × 1.0 cm. and the PIMSoft software was applied to the record, analyse, process and store the body surface flow maps. The changes in perfusion in each group were compared.

### 2.7 Post‐operative wound observation

The model and electroacupuncture groups were photographed in 1d, 4d and 7d using a digital camera, and trauma healing was analysed using image‐pro Plus 6.0 image analysis software. Percentage of trauma healing = [(original trauma area ‐ trauma area at the time of observation) ÷ original trauma area] × 100%.

### 2.8 Histological testing

Skin tissues of each group were taken from the moulded area after 7 days, fixed with 4% PFA solution for more than 24 h, dehydrated, and paraffin‐embedded to make 4 um sections. H&E and Masson staining were performed, and after completing the steps, the sections were dehydrated and sealed, observed under the microscope and photographed for comparison.

### 2.9 Statistical analysis

All of the experimental results were expressed as the mean ± SD (standard deviation). All statistical analyses were performed using SPSS 21.0 software. The significance of differences between groups was determined by 2‐tailed unpaired Student's *t*‐test or one‐way anova with Dunnett's post hoc test when samples were not distributed normally. A value of *p* < 0.05 was considered to be statistically significant.

## 3 RESULTS

### 3.1 Repeatability and stability of the UHPLC‐QTOF/MS method

**FIGURE 1:**
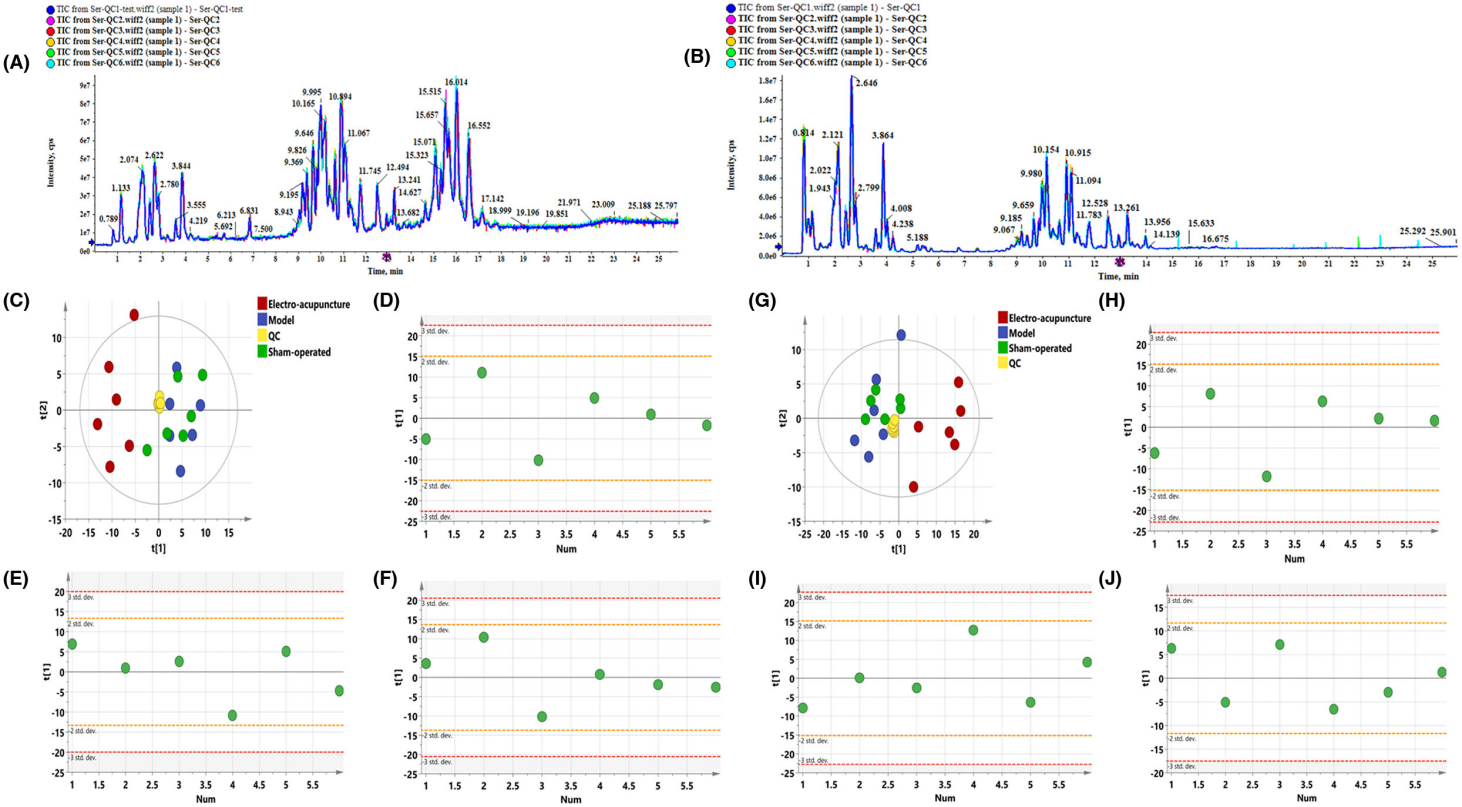
(A, B) The TIC diagram of QC samples in positive and negative ion mode. (A) Negative ion mode, (B) Positive ion mode. Figure 1 (C–F) PCA analysis of negative ions. (C) PCA analysis of all samples (R^2^X 0.770, Q^2^ 0.450). (D) PCA‐class analysis of sham‐operated group. (E) PCA‐class analysis of model group. (F) PCA‐class analysis of electro‐acupuncture group. Figure 1 (G–J) PCA analysis of positive ions. (G) PCA analysis of all samples (R^2^X 0.763, Q^2^ 0.547), (H) PCA‐class analysis of sham‐operated group. (I) PCA‐class analysis of model group. (J) PCA‐class analysis of electro‐acupuncture group.

Figure 1(A),1(B) show the QC Base Peak Chromatograms (BPC) in positive and negative ion modes. The Base Peak Chromatogram is a continuous depiction of the most intense ion intensity obtained at each time point, and the BPC contains the ion intensity as well as the retention time of the ion in the chromatogram. Figure 1(C), Figure 1(G) show the PCA plots of all samples in positive and negative ion mode, respectively. It can be seen that the QC samples are more tightly clustered, indicating that this experiment has good stability and reproducibility. The consistency of the samples within the group was analysed by PCA‐Class analysis, and all data could be retained as seen from the results in Figure 1 (C–F) and Figure 1 (G–J).

### 3.2 Results of differential metabolite analysis

#### 3.2.1 Post‐modelling affects 11 lipid metabolites in serum

**FIGURE 2:**
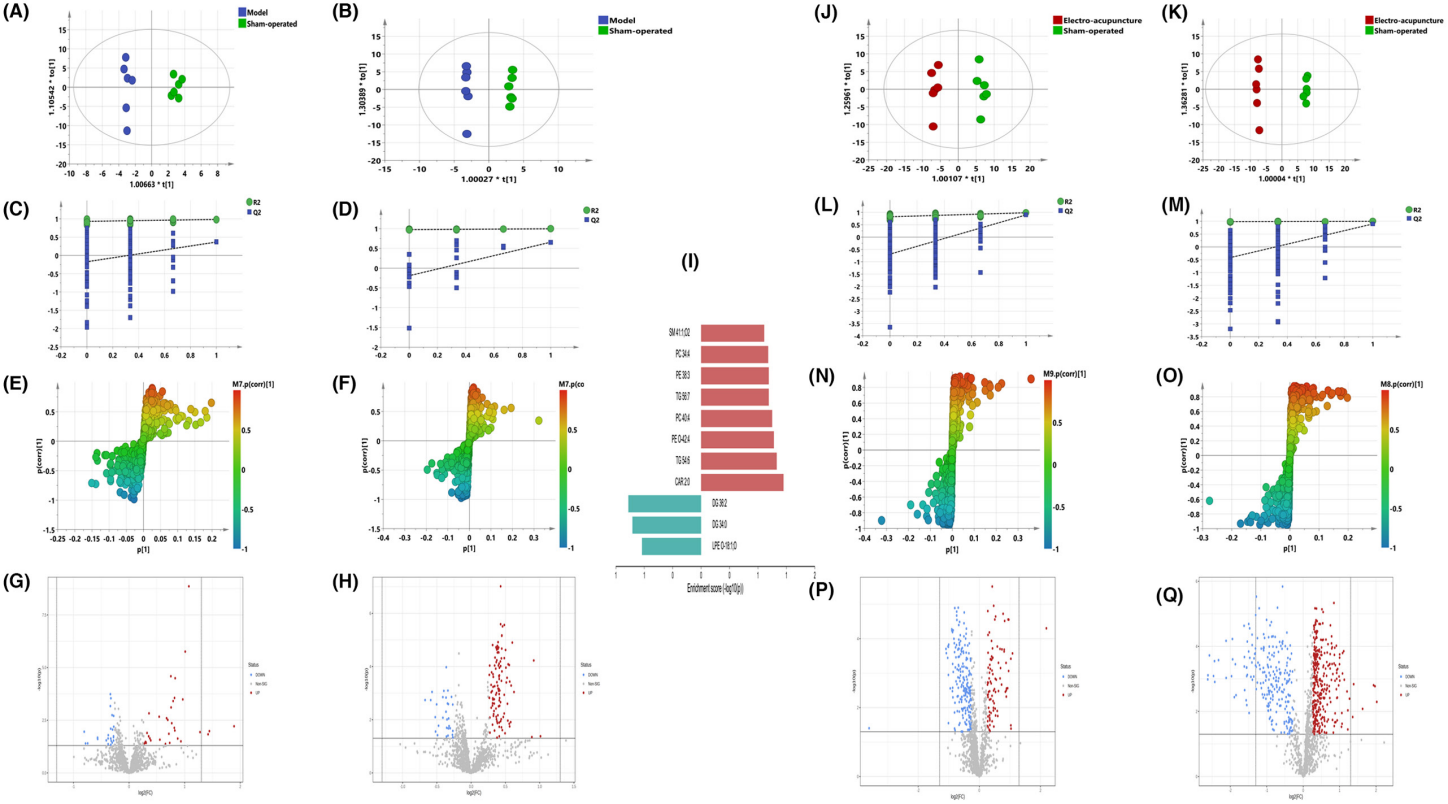
(A–F) OPLS‐DA: Model versus sham‐operated; (A) Negative ions (R^2^X 0.472, R^2^Y 0.984, Q^2^ 0.366). (B) Positive ions (R^2^X 0.776, R^2^Y 0.998, Q^2^ 0.658). (C) Negative ion replacement test. (D) Positive ion replacement tests. (E) Negative ion S‐plot. (F) Positive ion S‐plot. Figure 2 (G, H) Volcano diagram. (G) Negative ions. (H) Positive ions. ①Fold change (M/S) > 1.2 or <0.83, and ②*p* < 0.05 was the effective variable. Blue means down, red means up. Figure 2I model versus sham‐operated bar chart of differential metabolites. Red indicates an increase in the model group and green indicates a decrease in the model group. (J–O) OPLS‐DA: electro‐acupuncture versus sham‐operated; (J) Negative ions (R2X 0.702, R2Y 0.986, Q2 0.897). (K) Positive ions (R2X 0.878, R2Y 0.997, Q2 0.894). (l) Negative ion replacement test. (M) Positive ion replacement tests. (N) Negative ion S‐plot. (O) Positive ion S‐plot. Figure 2 (P, Q). Volcano diagram. (P) Negative ions. (Q) Positive ions. ①Fold change (E/S) > 1.2 or <0.83, and ②*p* < 0.05 was the effective variable. Blue means down, red means up.

Figure 2 (A–F) shows the OPLS‐DA plots for model versus sham‐operated, which shows that the two groups are clearly differentiated and the model is valid. Figure 2 (G–H) shows the volcano plot for model versus sham‐operated, with red colour indicating variables up‐regulated after modelling, blue colour indicating variables down‐regulated after modelling, and grey colour indicating variables with no differences. Eleven differential metabolites of model versus sham‐operated were screened and identified, and the results are shown in Table 1. Detailed response changes are shown in Figure 2I.

**TABLE 1:** Basic information of differential metabolites of model versus sham‐operated.

Name	Formula	Rt min	Ion	m/z	VIP	*p* (corr)	*p* Value	Fold change
CAR 2:0	C_9_H_17_NO_4_	0.75	M + H	204.1219	1.89	−0.70	0.0260	1.45
DG 34:0	C_37_H_72_O_5_	13.06	M + NH_4_	614.5725	1.48	0.59	0.0411	0.83
DG 36:2	C_39_H_72_O_5_	12.40	M + NH_4_	638.5734	1.14	0.65	0.0260	0.78
LPE O‐18:1;O	C_23_H_48_NO_7_P	2.62	M‐H	480.3071	2.53	0.62	0.0152	0.96
PC 34:4	C_42_H_76_NO_8_P	8.89	M + FA‐H	798.5227	2.31	−0.68	0.0152	1.18
PC 40:4	C_48_H_88_NO_8_P	11.74	M + FA‐H	882.6202	1.50	−0.69	0.0411	1.25
PE 38:3	C_43_H_80_NO_8_P	10.22	M‐H	768.5568	1.08	−0.62	0.0411	1.19
PE O‐42:4	C_47_H_88_NO_7_P	11.00	M + FA‐H	854.6225	1.26	−0.68	0.0411	1.28
SM 41:1;O2	C_46_H_93_N_2_O_6_P	12.92	M + FA‐H	845.6714	3.69	−0.54	0.0411	1.11
TG 54:6	C_57_H_98_O_6_	15.46	M + NH_4_	896.7688	1.61	−0.69	0.0043	1.33
TG 56:7	C_59_H_100_O_6_	15.59	M + NH_4_	922.7829	4.02	−0.63	0.0411	1.19

#### 3.2.2 Electroacupuncture affects 117 lipid metabolites in serum

**FIGURE 3:**
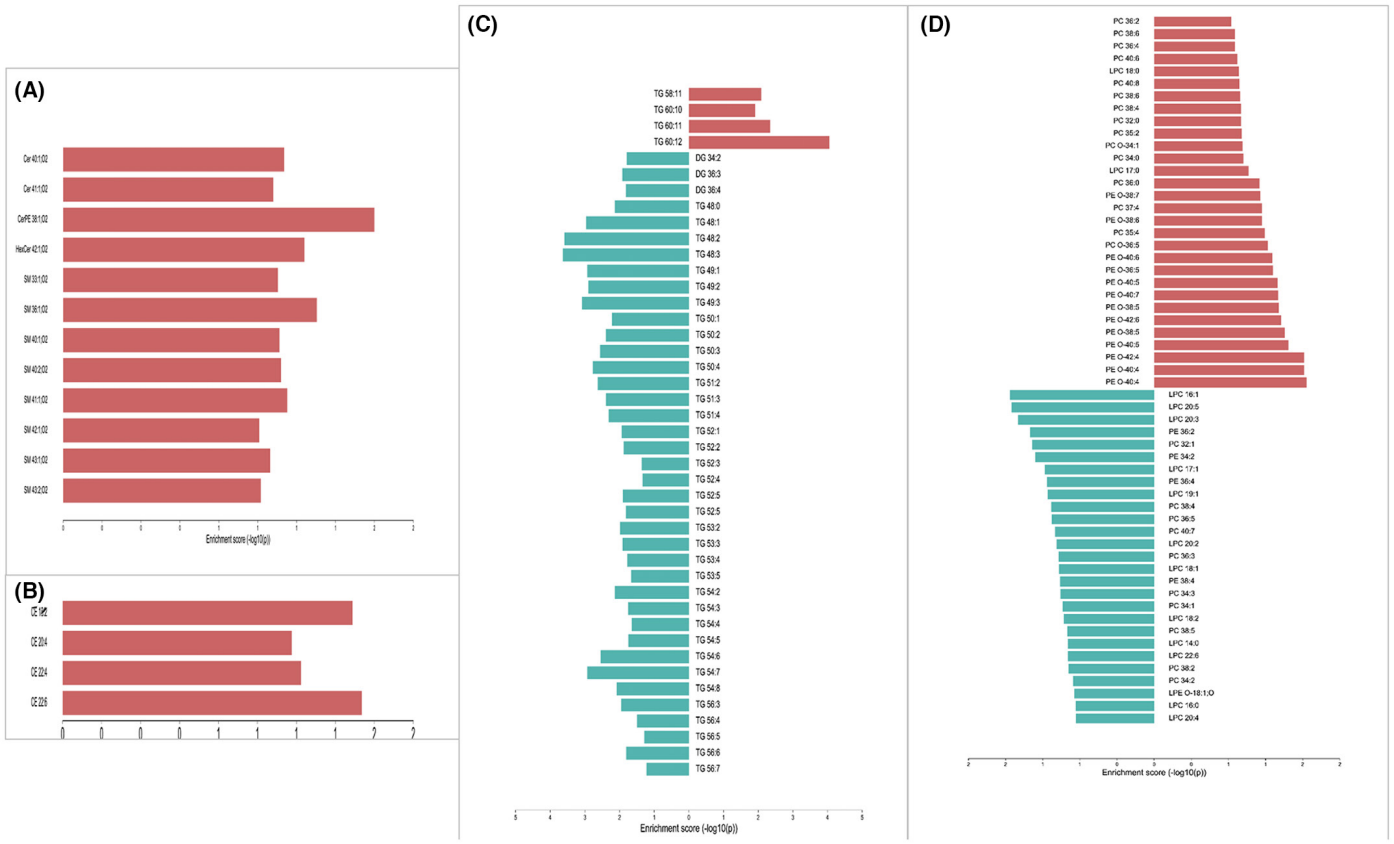
Bar chart of electro‐acupuncture versus sham‐operated differential metabolites. Red indicates an increase in the electroacupuncture group and green indicates a decrease in the electroacupuncture group. (A) Sphingolipids. (B) Cholesteryl esters. (C) Glycerides. (D) Glycerophospholipids.

Figure 2 (J–O) shows the OPLS‐DA plot for electro‐acupuncture versus sham‐operated, with a clear distinction between the two. Figure 2 (P,Q) shows the volcano plot for electro‐acupuncture versus sham‐operated, with red colour indicating variables upregulated after electro‐acupuncture, blue colour indicating variables downregulated after electro‐acupuncture, and grey colour indicating variables with no difference. The 117 differential metabolites of electro‐acupuncture versus sham‐operated were screened and identified, and it was seen that cholesteryl esters and sphingolipids were elevated after electro‐acupuncture, triglycerides were largely decreased after electro‐acupuncture with some triglycerides up‐regulated, and phospholipids showed no significant change pattern, the results are shown in Table 2. Detailed response changes are shown in Figure 3.

**TABLE 2:** Basic information of differential metabolites of electro‐acupuncture versus Sham‐operated.

Name	Formula	Rt min	Ion	m/z	VIP	*p* (corr)	*p* Value	Fold change
CAR 2:0	C_9_H_17_NO_4_	0.75	M + H	204.1219	1.21	−0.78	0.0152	1.66
CE 18:2	C_45_H_76_O_2_	15.98	M + NH_4_	666.6158	3.09	−0.87	0.0022	1.80
CE 20:4	C_47_H_76_O_2_	15.68	M + NH_4_	690.6132	5.84	−0.93	0.0022	1.39
CE 22:4	C_49_H_80_O_2_	16.14	M + NH_4_	718.6475	1.74	−0.80	0.0022	1.57
CE 22:6	C_49_H_76_O_2_	15.35	M + NH_4_	714.6165	2.56	−0.92	0.0022	1.72
Cer 40:1;O2	C_40_H_79_NO_3_	13.33	M + FA‐H	666.6015	1.53	−0.74	0.0260	1.42
Cer 41:1;O2	C_41_H_81_NO_3_	13.66	M + FA‐H	680.6162	1.74	−0.76	0.0087	1.35
CerPE 38:1;O2	C_40_H_81_N_2_O_6_P	10.40	M + FA‐H	761.5775	1.19	−0.86	0.0022	2.00
DG 34:2	C_37_H_68_O_5_	11.71	M + NH_4_	610.5434	1.09	0.67	0.0260	0.56
DG 36:3	C_39_H_70_O_5_	11.73	M + NH_4_	636.5554	1.46	0.74	0.0152	0.52
DG 36:4	C_39_H_68_O_5_	11.01	M + NH_4_	634.5416	1.17	0.64	0.0411	0.55
HexCer 42:1;O2	C_48_H_93_NO_8_	13.42	M + FA‐H	856.6847	1.66	−0.78	0.0087	1.55
LPC 14:0	C_22_H_46_NO_7_P	1.78	M + FA‐H	512.2970	2.07	0.77	0.0022	0.86
LPC 16:0	C_24_H_50_NO_7_P	2.62	M + FA‐H	540.3283	8.08	0.85	0.0022	0.95
LPC 16:1	C_24_H_48_NO_7_P	1.92	M + FA‐H	538.3134	6.20	0.84	0.0022	0.51
LPC 17:0	C_25_H_52_NO_7_P	3.2	M + H	510.3542	1.72	−0.57	0.0152	1.27
LPC 17:1	C_25_H_50_NO_7_P	2.33	M + FA‐H	552.3290	1.44	0.83	0.0087	0.68
LPC 18:0	C_26_H_54_NO_7_P	3.56	M + FA‐H	568.3595	4.25	−0.84	0.0087	1.14
LPC 18:1	C_26_H_52_NO_7_P	2.71	M + FA‐H	566.3492	2.98	0.81	0.0043	0.78
LPC 18:2	C_26_H_50_NO_7_P	2.13	M + FA‐H	564.3283	12.87	0.91	0.0022	0.82
LPC 19:1	C_27_H_54_NO_7_P	3.34	M + FA‐H	580.3628	1.06	0.81	0.0043	0.70
LPC 20:2	C_28_H_54_NO_7_P	3.03	M + FA‐H	592.3576	2.21	0.85	0.0022	0.76
LPC 20:3	C_28_H_52_NO_7_P	2.43	M + FA‐H	590.3457	5.50	0.86	0.0022	0.55
LPC 20:4	C_28_H_50_NO_7_P	2.05	M + FA‐H	588.3278	4.68	0.61	0.0411	0.95
LPC 20:5	C_28_H_48_NO_7_P	1.62	M + FA‐H	586.3144	2.98	0.90	0.0022	0.52
LPC 22:6	C_30_H_50_NO_7_P	1.9	M + FA‐H	612.3289	3.44	0.79	0.0260	0.86
LPE O‐18:1;O	C_23_H_48_NO_7_P	2.62	M‐H	480.3071	2.00	0.81	0.0043	0.93
PC 32:0	C_40_H_80_NO_8_P	10.86	M + FA‐H	778.5571	2.45	−0.77	0.0087	1.17
PC 32:1	C_40_H_78_NO_8_P	9.95	M + H	732.5520	2.66	0.74	0.0087	0.61
PC 34:0	C_42_H_84_NO_8_P	11.73	M + FA‐H	806.5884	2.15	−0.76	0.0152	1.20
PC 34:1	C_42_H_82_NO_8_P	10.89	M + FA‐H	804.5726	5.73	0.76	0.0087	0.81
PC 34:2	C_42_H_80_NO_8_P	10.13	M + FA‐H	802.5553	6.30	0.70	0.0411	0.91
PC 34:3	C_42_H_78_NO_8_P	9.17	M + FA‐H	800.5409	2.47	0.83	0.0022	0.79
PC 35:2	C_43_H_82_NO_8_P	10.63	M + H	772.5835	2.16	−0.54	0.0411	1.18
PC 35:4	C_43_H_78_NO_8_P	9.43	M + H	768.5561	1.79	−0.58	0.0260	1.49
PC 36:0	C_44_H_88_NO_8_P	12.50	M + FA‐H	834.6222	1.52	−0.93	0.0022	1.42
PC 36:2	C_44_H_84_NO_8_P	11.09	M + H	786.5969	2.78	−0.78	0.0087	1.04
PC 36:3	C_44_H_82_NO_8_P	10.36	M + FA‐H	828.5711	6.54	0.81	0.0087	0.78
PC 36:4	C_44_H_80_NO_8_P	9.94	M + H	782.5660	4.17	−0.84	0.0022	1.09
PC 36:5	C_44_H_78_NO_8_P	9.23	M + FA‐H	824.5439	1.78	0.69	0.0260	0.73
PC 37:4	C_45_H_82_NO_8_P	10.46	M + FA‐H	840.5739	3.04	−0.63	0.0260	1.45
PC 38:2	C_46_H_88_NO_8_P	11.84	M + FA‐H	858.6198	1.85	0.68	0.0260	0.87
PC 38:4	C_46_H_84_NO_8_P	10.92	M + FA‐H	854.5866	7.74	−0.70	0.0260	1.17
PC 38:4	C_46_H_84_NO_8_P	10.33	M + FA‐H	854.5860	3.07	0.91	0.0022	0.72
PC 38:5	C_46_H_82_NO_8_P	10.00	M + FA‐H	852.5739	2.73	0.69	0.0152	0.86
PC 38:6	C_46_H_80_NO_8_P	9.63	M + H	806.5649	3.49	−0.89	0.0022	1.09
PC 38:6	C_46_H_80_NO_8_P	9.19	M + H	806.5679	3.16	−0.71	0.0260	1.16
PC 40:6	C_48_H_84_NO_8_P	10.64	M + FA‐H	878.5869	3.48	−0.67	0.0260	1.12
PC 40:7	C_48_H_82_NO_8_P	9.7	M + FA‐H	876.5757	1.94	0.85	0.0043	0.75
PC 40:8	C_48_H_80_NO_8_P	8.94	M + FA‐H	874.5584	1.84	−0.75	0.0087	1.15
PC O‐34:1	C_42_H_84_NO_7_P	11.43	M + FA‐H	790.5953	1.08	−0.75	0.0152	1.19
PC O‐36:5	C_44_H_80_NO_7_P	10.38	M + FA‐H	810.5664	1.48	−0.85	0.0043	1.53
PE 34:2	C_39_H_74_NO_8_P	10.42	M‐H	714.5078	1.36	0.85	0.0022	0.63
PE 36:2	C_41_H_78_NO_8_P	11.34	M‐H	742.5356	2.07	0.86	0.0022	0.60
PE 36:4	C_41_H_74_NO_8_P	10.22	M‐H	738.5123	1.01	0.73	0.0087	0.69
PE 38:4	C_43_H_78_NO_8_P	11.16	M‐H	766.5366	1.42	0.61	0.0411	0.79
PE O‐36:5	C_41_H_74_NO_7_P	10.67	M‐H	722.5137	1.57	−0.86	0.0022	1.60
PE O‐38:5	C_43_H_78_NO_7_P	11.58	M‐H	750.5406	2.17	−0.90	0.0022	1.68
PE O‐38:5	C_43_H_78_NO_7_P	11.33	M‐H	750.5447	1.24	−0.89	0.0022	1.76
PE O‐38:6	C_43_H_76_NO_7_P	10.72	M‐H	748.5301	1.56	−0.87	0.0022	1.45
PE O‐38:7	C_43_H_74_NO_7_P	10.37	M‐H	746.5078	1.08	−0.83	0.0022	1.43
PE O‐40:4	C_45_H_84_NO_7_P	12.26	M‐H	780.5909	1.32	−0.91	0.0022	2.02
PE O‐40:4	C_45_H_84_NO_7_P	12.47	M‐H	780.5890	1.22	−0.88	0.0022	2.05
PE O‐40:5	C_45_H_82_NO_7_P	12.15	M‐H	778.5738	1.49	−0.94	0.0022	1.81
PE O‐40:5	C_45_H_82_NO_7_P	12.37	M‐H	778.5716	1.17	−0.94	0.0022	1.66
PE O‐40:6	C_45_H_80_NO_7_P	11.57	M‐H	776.5602	1.35	−0.92	0.0022	1.59
PE O‐40:7	C_45_H_78_NO_7_P	11.28	M‐H	774.5435	1.13	−0.94	0.0022	1.67
PE O‐42:4	C_47_H_88_NO_7_P	12.95	M‐H	808.6168	1.01	−0.85	0.0043	2.02
PE O‐42:6	C_47_H_84_NO_7_P	12.34	M‐H	804.5895	1.06	−0.90	0.0022	1.71
SM 33:1;O2	C_38_H_77_N_2_O_6_P	9.26	M + H	689.5599	1.33	−0.83	0.0043	1.38
SM 36:1;O2	C_41_H_83_N_2_O_6_P	10.88	M + FA‐H	775.5962	1.55	−0.85	0.0022	1.63
SM 40:1;O2	C_45_H_91_N_2_O_6_P	12.59	M + FA‐H	831.6596	3.96	−0.89	0.0043	1.39
SM 40:2;O2	C_45_H_89_N_2_O_6_P	11.73	M + H	785.6541	1.49	−0.78	0.0043	1.40
SM 41:1;O2	C_46_H_93_N_2_O_6_P	12.92	M + FA‐H	845.6714	4.35	−0.75	0.0022	1.44
SM 42:1;O2	C_47_H_95_N_2_O_6_P	13.26	M + FA‐H	859.6842	5.99	−0.82	0.0043	1.26
SM 43:1;O2	C_48_H_97_N_2_O_6_P	13.46	M + FA‐H	873.7043	2.53	−0.76	0.0043	1.33
SM 43:2;O2	C_48_H_95_N_2_O_6_P	12.84	M + FA‐H	871.6880	1.32	−0.66	0.0152	1.27
TG 48:0	C_51_H_98_O_6_	16.62	M + NH_4_	824.7692	1.48	0.88	0.0022	0.47
TG 48:1	C_51_H_96_O_6_	16.00	M + NH_4_	822.7537	2.08	0.75	0.0087	0.34
TG 48:2	C_51_H_94_O_6_	15.42	M + NH_4_	820.7375	2.63	0.77	0.0043	0.28
TG 48:3	C_51_H_92_O_6_	14.94	M + NH_4_	818.7248	1.39	0.86	0.0022	0.27
TG 49:1	C_52_H_98_O_6_	16.26	M + NH_4_	836.7679	1.39	0.85	0.0022	0.34
TG 49:2	C_52_H_96_O_6_	15.72	M + NH_4_	834.7527	2.00	0.90	0.0022	0.35
TG 49:3	C_52_H_94_O_6_	15.22	M + NH_4_	832.7388	1.17	0.92	0.0022	0.32
TG 50:1	C_53_H_100_O_6_	16.57	M + NH_4_	850.7825	3.92	0.79	0.0087	0.45
TG 50:2	C_53_H_98_O_6_	15.97	M + NH_4_	848.7664	6.43	0.81	0.0022	0.42
TG 50:3	C_53_H_96_O_6_	15.47	M + NH_4_	846.7517	5.12	0.83	0.0043	0.39
TG 50:4	C_53_H_94_O_6_	14.96	M + NH_4_	844.7417	2.83	0.92	0.0022	0.36
TG 51:2	C_54_H_100_O_6_	16.24	M + NH_4_	862.7850	2.53	0.82	0.0022	0.38
TG 51:3	C_54_H_98_O_6_	15.71	M + NH_4_	860.7677	2.70	0.91	0.0022	0.42
TG 51:4	C_54_H_96_O_6_	15.26	M + NH_4_	858.7551	1.97	0.94	0.0022	0.43
TG 52:1	C_55_H_104_O_6_	17.18	M + NH_4_	878.8185	1.64	0.71	0.0152	0.52
TG 52:2	C_55_H_102_O_6_	16.56	M + NH_4_	876.7966	7.35	0.79	0.0087	0.53
TG 52:3	C_55_H_100_O_6_	15.99	M + NH_4_	874.7787	6.55	0.86	0.0043	0.74
TG 52:4	C_55_H_98_O_6_	15.51	M + NH_4_	872.7644	5.93	0.87	0.0043	0.75
TG 52:5	C_55_H_96_O_6_	15.09	M + NH_4_	870.7541	4.54	0.89	0.0022	0.52
TG 52:5	C_55_H_96_O_6_	15.95	M + H	853.7278	1.25	0.78	0.0087	0.55
TG 53:2	C_56_H_104_O_6_	16.85	M + NH_4_	890.8129	1.10	0.77	0.0087	0.50
TG 53:3	C_56_H_102_O_6_	16.32	M + NH_4_	888.7928	1.82	0.84	0.0022	0.52
TG 53:4	C_56_H_100_O_6_	15.79	M + NH_4_	886.7815	1.72	0.90	0.0022	0.56
TG 53:5	C_56_H_98_O_6_	15.24	M + NH_4_	884.7687	1.08	0.89	0.0022	0.60
TG 54:2	C_57_H_106_O_6_	17.12	M + NH_4_	904.8272	2.05	0.76	0.0087	0.47
TG 54:3	C_57_H_104_O_6_	16.57	M + NH_4_	902.8122	4.09	0.83	0.0022	0.57
TG 54:4	C_57_H_102_O_6_	15.99	M + NH_4_	900.7970	5.25	0.84	0.0022	0.61
TG 54:5	C_57_H_100_O_6_	15.52	M + NH_4_	898.7803	5.89	0.88	0.0022	0.57
TG 54:6	C_57_H_98_O_6_	15.04	M + NH_4_	896.7657	6.28	0.87	0.0022	0.39
TG 54:7	C_57_H_96_O_6_	14.62	M + NH_4_	894.7518	2.94	0.85	0.0043	0.34
TG 54:8	C_57_H_94_O_6_	14.59	M + NH_4_	892.7418	1.32	0.91	0.0022	0.48
TG 56:3	C_59_H_108_O_6_	17.15	M + NH_4_	930.8400	1.22	0.71	0.0087	0.51
TG 56:4	C_59_H_106_O_6_	16.52	M + NH_4_	928.8289	1.69	0.76	0.0087	0.67
TG 56:5	C_59_H_104_O_6_	16.26	M + NH_4_	926.8127	2.42	0.76	0.0087	0.78
TG 56:6	C_59_H_102_O_6_	15.02	M + NH_4_	924.7995	1.53	0.88	0.0022	0.55
TG 56:7	C_59_H_100_O_6_	15.59	M + NH_4_	922.7829	2.11	0.84	0.0022	0.82
TG 58:11	C_61_H_96_O_6_	14.31	M + NH_4_	942.7602	1.07	−0.70	0.0152	2.09
TG 60:10	C_63_H_102_O_6_	15.09	M + NH_4_	972.8038	1.49	−0.77	0.0087	1.91
TG 60:11	C_63_H_100_O_6_	14.85	M + NH_4_	970.7877	1.32	−0.72	0.0411	2.34
TG 60:12	C_63_H_98_O_6_	14.47	M + NH_4_	968.7703	1.70	−0.75	0.0043	4.05

#### 3.2.3 Electro‐acupuncture and modelling can trigger different lipid metabolites in serum

**FIGURE 4:**
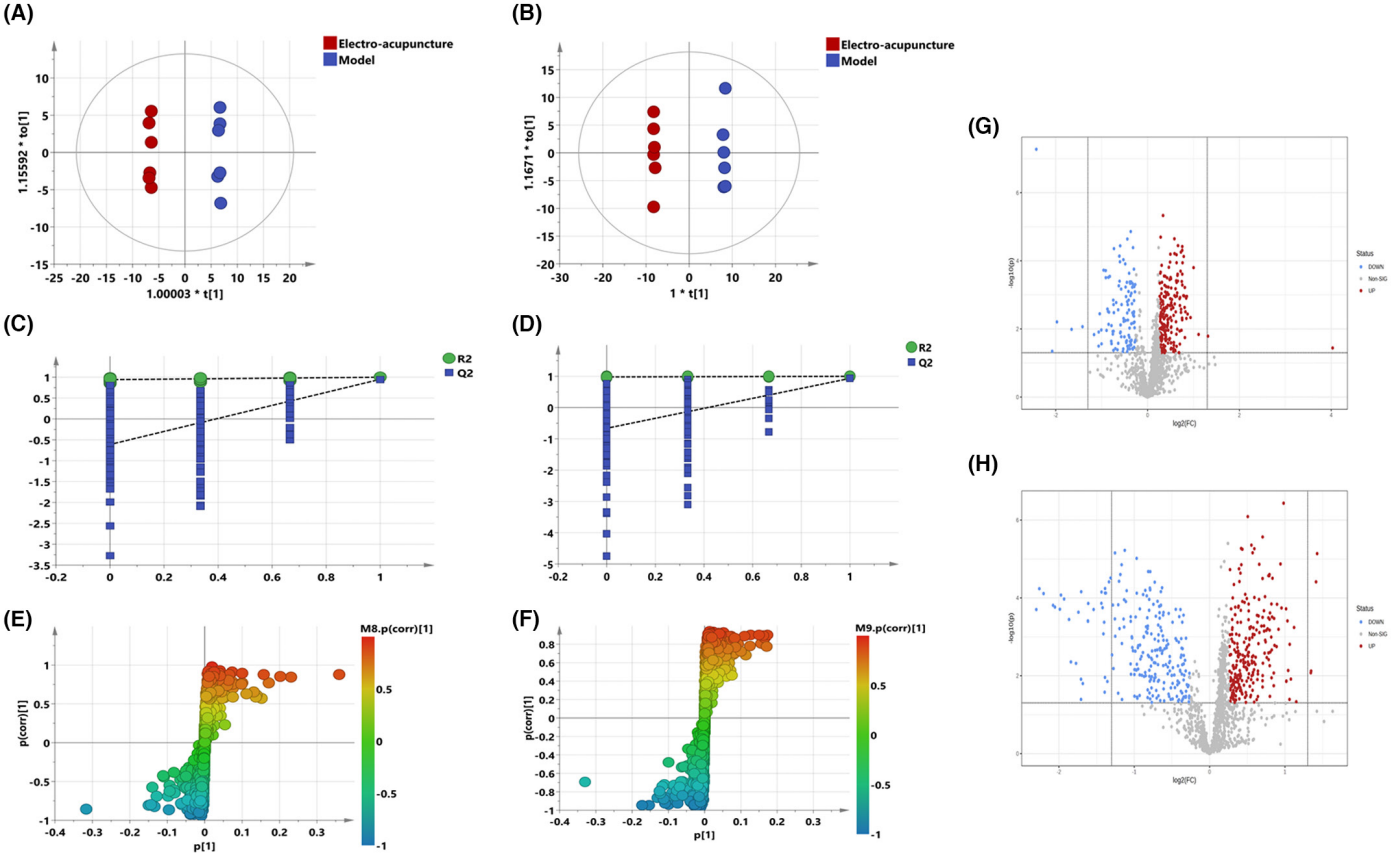
(A–F) OPLS‐DA: Electro‐acupuncture versus Model; (A) Negative ions (R^2^X 0.736, R^2^Y 0.999, Q^2^ 0.949). (B) Positive ions (R^2^X 0.892, R^2^Y 1.000, Q^2^ 0.933). (C) Negative ion replacement test. (D) Positive ion replacement tests. (E) Negative ion S‐plot. (F) Positive ion S‐plot. Figure 5 (G, H). Volcano diagram. (G) Negative ions. (H) Positive ions. ①Fold change (E/M) > 1.2 or <0.83, and ②*p* < 0.05 was the effective variable. Blue means down, red means up.

**FIGURE 5:**
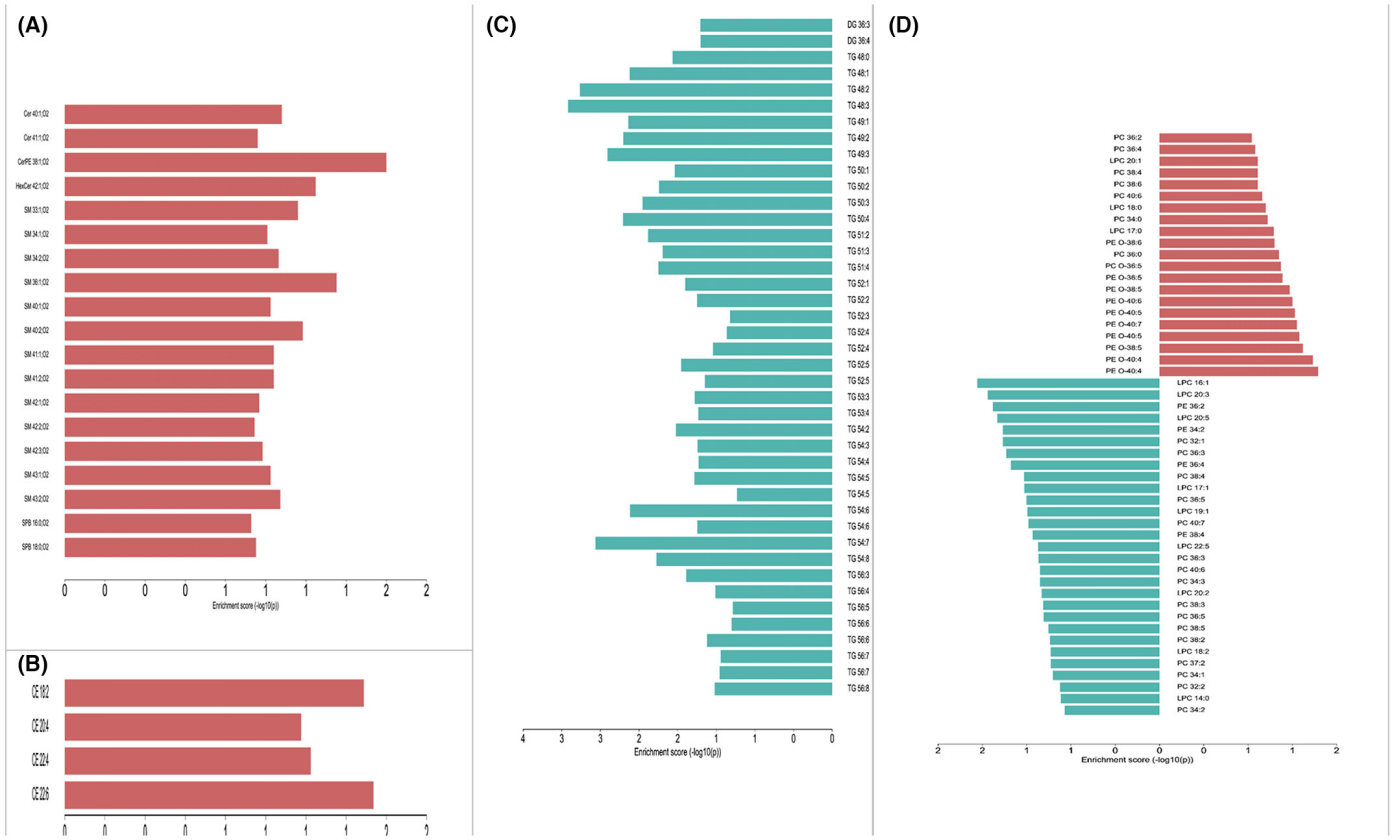
Bar chart of electro‐acupuncture versus Model differential metabolites. Red indicates an increase in the electroacupuncture group and green indicates a decrease in the electroacupuncture group. (A) Sphingolipids. (B) Cholesteryl esters. (C) Glycerides. (D) Glycerophospholipids.

Figure 4 (A–F) shows the OPLS‐DA plots for electro‐acupuncture versus model, which shows that the two groups are clearly differentiated and the model is valid. Figure 4 (G–H) shows the volcano plot for electro‐acupuncture versus model, with red colour indicating variables that were upregulated after electroacupuncture, blue colour indicating variables that were downregulated after electroacupuncture, and grey colour indicating variables with no difference. The 115 differential metabolites of the electro‐acupuncture versus model were screened and identified, and the pattern of differential metabolite changes was consistent with the results of 3.2.2, and the results are shown in Table 3. Detailed response changes are shown in Figure 5.

**TABLE 3:** Basic information of differential metabolites of electro‐acupuncture versus model.

Name	Formula	Rt min	Ion	m/z	VIP	*p* (corr)	*p* Value	Fold change
CE 18:2	C_45_H_76_O_2_	15.98	M + NH_4_	666.6158	2.96	−0.88	0.0022	1.86
CE 20:4	C_47_H_76_O_2_	15.68	M + NH_4_	690.6132	5.87	−0.94	0.0022	1.47
CE 22:4	C_49_H_80_O_2_	16.14	M + NH_4_	718.6475	1.64	−0.77	0.0087	1.53
CE 22:6	C_49_H_76_O_2_	15.35	M + NH_4_	714.6165	2.56	−0.93	0.0022	1.92
Cer 40:1;O2	C_40_H_79_NO_3_	13.33	M + FA‐H	666.6015	1.40	−0.74	0.0152	1.35
Cer 41:1;O2	C_41_H_81_NO_3_	13.66	M + FA‐H	680.6162	1.22	−0.61	0.0411	1.20
CerPE 38:1;O2	C_40_H_81_N_2_O_6_P	10.40	M + FA‐H	761.5775	1.19	−0.88	0.0022	2.00
DG 36:3	C_39_H_70_O_5_	11.73	M + NH_4_	636.5554	1.20	0.65	0.0411	0.59
DG 36:4	C_39_H_68_O_5_	11.01	M + NH_4_	634.5416	1.05	0.58	0.0260	0.59
HexCer 42:1;O2	C_48_H_93_NO_8_	13.42	M + FA‐H	856.6847	1.65	−0.81	0.0087	1.56
LPC 14:0	C_22_H_46_NO_7_P	1.78	M + FA‐H	512.297	1.71	0.78	0.0087	0.90
LPC 16:1	C_24_H_48_NO_7_P	1.92	M + FA‐H	538.3134	6.35	0.77	0.0022	0.49
LPC 17:0	C_25_H_52_NO_7_P	3.2	M + H	510.3542	1.68	−0.56	0.0260	1.29
LPC 17:1	C_25_H_50_NO_7_P	2.33	M + FA‐H	552.329	1.50	0.85	0.0022	0.65
LPC 18:0	C_26_H_54_NO_7_P	3.56	M + FA‐H	568.3595	4.78	−0.82	0.0043	1.20
LPC 18:2	C_26_H_50_NO_7_P	2.13	M + FA‐H	564.3283	12.79	0.88	0.0022	0.81
LPC 19:1	C_27_H_54_NO_7_P	3.34	M + FA‐H	580.3628	1.14	0.84	0.0022	0.67
LPC 20:1	C_28_H_56_NO_7_P	3.98	M + FA‐H	594.3771	1.08	−0.59	0.0260	1.11
LPC 20:2	C_28_H_54_NO_7_P	3.03	M + FA‐H	592.3576	2.13	0.80	0.0043	0.75
LPC 20:3	C_28_H_52_NO_7_P	2.43	M + FA‐H	590.3457	5.68	0.88	0.0022	0.52
LPC 20:5	C_28_H_48_NO_7_P	1.62	M + FA‐H	586.3144	2.74	0.78	0.0022	0.55
LPC 22:5	C_30_H_52_NO_7_P	2.42	M + FA‐H	614.3463	1.07	0.53	0.0411	0.73
PC 32:1	C_40_H_78_NO_8_P	9.95	M + H	732.552	2.74	0.65	0.0152	0.57
PC 32:2	C_40_H_76_NO_8_P	9.07	M + FA‐H	774.5251	1.48	0.71	0.0152	0.89
PC 34:0	C_42_H_84_NO_8_P	11.73	M + FA‐H	806.5884	2.23	−0.80	0.0043	1.22
PC 34:1	C_42_H_82_NO_8_P	10.89	M + FA‐H	804.5726	5.21	0.65	0.0152	0.83
PC 34:2	C_42_H_80_NO_8_P	10.13	M + FA‐H	802.5553	5.48	0.59	0.0411	0.93
PC 34:3	C_42_H_78_NO_8_P	9.17	M + FA‐H	800.5409	2.79	0.75	0.0022	0.74
PC 36:0	C_44_H_88_NO_8_P	12.50	M + FA‐H	834.6222	1.35	−0.83	0.0022	1.35
PC 36:2	C_44_H_84_NO_8_P	11.09	M + H	786.5969	2.49	−0.69	0.0260	1.04
PC 36:3	C_44_H_82_NO_8_P	10.36	M + FA‐H	828.5711	7.33	0.85	0.0022	0.73
PC 36:3	C_44_H_82_NO_8_P	10.57	M + FA‐H	828.5763	1.77	0.66	0.0411	0.58
PC 36:4	C_44_H_80_NO_8_P	9.94	M + H	782.566	3.73	−0.87	0.0022	1.08
PC 36:5	C_44_H_78_NO_8_P	9.23	M + FA‐H	824.5439	2.04	0.73	0.0043	0.67
PC 36:5	C_44_H_78_NO_8_P	8.99	M + FA‐H	824.5422	1.92	0.57	0.0411	0.76
PC 37:2	C_45_H_86_NO_8_P	11.49	M + FA‐H	844.6031	2.05	0.75	0.0043	0.81
PC 38:2	C_46_H_88_NO_8_P	11.84	M + FA‐H	858.6198	2.32	0.73	0.0087	0.81
PC 38:3	C_46_H_86_NO_8_P	11.30	M + FA‐H	856.6046	4.66	0.63	0.0260	0.76
PC 38:4	C_46_H_84_NO_8_P	10.92	M + FA‐H	854.5866	5.85	−0.58	0.0411	1.11
PC 38:4	C_46_H_84_NO_8_P	10.33	M + FA‐H	854.586	3.55	0.90	0.0022	0.65
PC 38:5	C_46_H_82_NO_8_P	10.00	M + FA‐H	852.5739	3.78	0.77	0.0087	0.80
PC 38:6	C_46_H_80_NO_8_P	9.63	M + H	806.5649	3.65	−0.93	0.0022	1.11
PC 40:6	C_48_H_84_NO_8_P	10.64	M + FA‐H	878.5869	3.58	−0.62	0.0260	1.16
PC 40:6	C_48_H_84_NO_8_P	10.13	M + FA‐H	878.5936	1.38	0.80	0.0043	0.74
PC 40:7	C_48_H_82_NO_8_P	9.7	M + FA‐H	876.5757	2.31	0.80	0.0043	0.68
PC O‐36:5	C_44_H_80_NO_7_P	10.38	M + FA‐H	810.5664	1.26	−0.83	0.0043	1.37
PE 34:2	C_39_H_74_NO_8_P	10.42	M‐H	714.5078	1.54	0.87	0.0022	0.57
PE 36:2	C_41_H_78_NO_8_P	11.34	M‐H	742.5356	2.36	0.87	0.0022	0.53
PE 36:4	C_41_H_74_NO_8_P	10.22	M‐H	738.5123	1.29	0.81	0.0022	0.60
PE 38:4	C_43_H_78_NO_8_P	11.16	M‐H	766.5366	1.94	0.74	0.0087	0.70
PE O‐36:5	C_41_H_74_NO_7_P	10.67	M‐H	722.5137	1.19	−0.66	0.0411	1.39
PE O‐38:5	C_43_H_78_NO_7_P	11.58	M‐H	750.5406	1.82	−0.79	0.0043	1.47
PE O‐38:5	C_43_H_78_NO_7_P	11.33	M‐H	750.5447	1.09	−0.82	0.0043	1.62
PE O‐38:6	C_43_H_76_NO_7_P	10.72	M‐H	748.5301	1.21	−0.72	0.0087	1.30
PE O‐40:4	C_45_H_84_NO_7_P	12.26	M‐H	780.5909	1.18	−0.90	0.0022	1.73
PE O‐40:4	C_45_H_84_NO_7_P	12.47	M‐H	780.589	1.08	−0.82	0.0043	1.79
PE O‐40:5	C_45_H_82_NO_7_P	12.15	M‐H	778.5738	1.31	−0.92	0.0022	1.58
PE O‐40:5	C_45_H_82_NO_7_P	12.37	M‐H	778.5716	1.03	−0.84	0.0022	1.53
PE O‐40:6	C_45_H_80_NO_7_P	11.57	M‐H	776.5602	1.24	−0.91	0.0022	1.50
PE O‐40:7	C_45_H_78_NO_7_P	11.28	M‐H	774.5435	1.01	−0.89	0.0022	1.55
SM 33:1;O2	C_38_H_77_N_2_O_6_P	9.26	M + H	689.5599	1.32	−0.84	0.0022	1.45
SM 34:1;O2	C_39_H_79_N_2_O_6_P	9.85	M + FA‐H	747.5638	5.35	−0.81	0.0022	1.26
SM 34:2;O2	C_39_H_77_N_2_O_6_P	8.79	M + H	701.5589	1.09	−0.64	0.0411	1.33
SM 36:1;O2	C_41_H_83_N_2_O_6_P	10.88	M + FA‐H	775.5962	1.62	−0.86	0.0022	1.69
SM 40:1;O2	C_45_H_91_N_2_O_6_P	12.59	M + FA‐H	831.6596	3.44	−0.86	0.0043	1.28
SM 40:2;O2	C_45_H_89_N_2_O_6_P	11.73	M + H	785.6541	1.46	−0.77	0.0087	1.48
SM 41:1;O2	C_46_H_93_N_2_O_6_P	12.92	M + FA‐H	845.6714	3.65	−0.69	0.0087	1.30
SM 41:2;O2	C_46_H_91_N_2_O_6_P	12.25	M + FA‐H	843.6561	1.93	−0.57	0.0260	1.30
SM 42:1;O2	C_47_H_95_N_2_O_6_P	13.26	M + FA‐H	859.6842	5.23	−0.80	0.0260	1.21
SM 42:2;O2	C_47_H_93_N_2_O_6_P	12.50	M + FA‐H	857.6718	4.99	−0.69	0.0260	1.18
SM 42:3;O2	C_47_H_91_N_2_O_6_P	11.79	M + FA‐H	855.6544	3.13	−0.65	0.0260	1.23
SM 43:1;O2	C_48_H_97_N_2_O_6_P	13.46	M + FA‐H	873.7043	2.27	−0.78	0.0087	1.28
SM 43:2;O2	C_48_H_95_N_2_O_6_P	12.84	M + FA‐H	871.688	1.43	−0.76	0.0043	1.34
SPB 16:0;O2	C_16_H_35_NO_2_	1.13	M + H	274.2719	2.10	−0.52	0.0411	1.16
SPB 18:0;O2	C_18_H_39_NO_2_	1.52	M + H	302.3032	1.04	−0.67	0.0152	1.19
TG 48:0	C_51_H_98_O_6_	16.62	M + NH_4_	824.7692	1.33	0.86	0.0022	0.48
TG 48:1	C_51_H_96_O_6_	16.00	M + NH_4_	822.7537	1.79	0.70	0.0152	0.38
TG 48:2	C_51_H_94_O_6_	15.42	M + NH_4_	820.7375	2.31	0.69	0.0043	0.31
TG 48:3	C_51_H_92_O_6_	14.94	M + NH_4_	818.7248	1.25	0.75	0.0043	0.29
TG 49:1	C_52_H_98_O_6_	16.26	M + NH_4_	836.7679	1.19	0.85	0.0022	0.38
TG 49:2	C_52_H_96_O_6_	15.72	M + NH_4_	834.7527	1.77	0.90	0.0022	0.37
TG 49:3	C_52_H_94_O_6_	15.22	M + NH_4_	832.7388	1.04	0.87	0.0022	0.34
TG 50:1	C_53_H_100_O_6_	16.57	M + NH_4_	850.7825	3.42	0.80	0.0087	0.49
TG 50:2	C_53_H_98_O_6_	15.97	M + NH_4_	848.7664	5.70	0.81	0.0022	0.45
TG 50:3	C_53_H_96_O_6_	15.47	M + NH_4_	846.7517	4.66	0.78	0.0043	0.41
TG 50:4	C_53_H_94_O_6_	14.96	M + NH_4_	844.7417	2.61	0.88	0.0022	0.37
TG 51:2	C_54_H_100_O_6_	16.24	M + NH_4_	862.785	2.17	0.82	0.0043	0.42
TG 51:3	C_54_H_98_O_6_	15.71	M + NH_4_	860.7677	2.30	0.89	0.0022	0.46
TG 51:4	C_54_H_96_O_6_	15.26	M + NH_4_	858.7551	1.78	0.93	0.0022	0.44
TG 52:1	C_55_H_104_O_6_	17.18	M + NH_4_	878.8185	1.49	0.71	0.0152	0.53
TG 52:2	C_55_H_102_O_6_	16.56	M + NH_4_	876.7966	6.36	0.77	0.0087	0.57
TG 52:3	C_55_H_100_O_6_	15.99	M + NH_4_	874.7787	5.87	0.86	0.0022	0.76
TG 52:4	C_55_H_98_O_6_	15.51	M + NH_4_	872.7644	5.92	0.88	0.0043	0.73
TG 52:4	C_55_H_98_O_6_	15.86	M + NH_4_	872.7717	1.24	0.79	0.0087	0.65
TG 52:5	C_55_H_96_O_6_	15.09	M + NH_4_	870.7541	4.46	0.90	0.0022	0.51
TG 52:5	C_55_H_96_O_6_	15.95	M + H	853.7278	1.04	0.75	0.0087	0.61
TG 53:3	C_56_H_102_O_6_	16.32	M + NH_4_	888.7928	1.55	0.83	0.0043	0.56
TG 53:4	C_56_H_100_O_6_	15.79	M + NH_4_	886.7815	1.57	0.90	0.0022	0.58
TG 54:2	C_57_H_106_O_6_	17.12	M + NH_4_	904.8272	1.82	0.79	0.0087	0.49
TG 54:3	C_57_H_104_O_6_	16.57	M + NH_4_	902.8122	3.84	0.89	0.0022	0.57
TG 54:4	C_57_H_102_O_6_	15.99	M + NH_4_	900.797	5.26	0.90	0.0022	0.58
TG 54:5	C_57_H_100_O_6_	15.52	M + NH_4_	898.7803	5.69	0.89	0.0022	0.56
TG 54:5	C_57_H_100_O_6_	15.85	M + NH_4_	898.7812	2.10	0.67	0.0152	0.81
TG 54:6	C_57_H_98_O_6_	15.04	M + NH_4_	896.7657	6.07	0.90	0.0022	0.38
TG 54:6	C_57_H_98_O_6_	15.46	M + NH_4_	896.7688	1.08	0.88	0.0022	0.57
TG 54:7	C_57_H_96_O_6_	14.62	M + NH_4_	894.7518	2.85	0.85	0.0022	0.33
TG 54:8	C_57_H_94_O_6_	14.59	M + NH_4_	892.7418	1.35	0.92	0.0022	0.44
TG 56:3	C_59_H_108_O_6_	17.15	M + NH_4_	930.84	1.11	0.76	0.0087	0.53
TG 56:4	C_59_H_106_O_6_	16.52	M + NH_4_	928.8289	1.60	0.79	0.0043	0.66
TG 56:5	C_59_H_104_O_6_	16.26	M + NH_4_	926.8127	2.34	0.74	0.0087	0.78
TG 56:6	C_59_H_102_O_6_	15.80	M + NH_4_	924.7982	3.09	0.77	0.0022	0.77
TG 56:6	C_59_H_102_O_6_	15.02	M + NH_4_	924.7995	1.25	0.79	0.0043	0.62
TG 56:7	C_59_H_100_O_6_	15.31	M + NH_4_	922.7825	3.86	0.72	0.0043	0.69
TG 56:7	C_59_H_100_O_6_	15.59	M + NH_4_	922.7829	2.93	0.87	0.0022	0.69
TG 56:8	C_59_H_98_O_6_	15.12	M + NH_4_	920.7671	4.47	0.68	0.0152	0.66

#### 3.2.4 Different metabolite profiles in different groups

**FIGURE 6:**
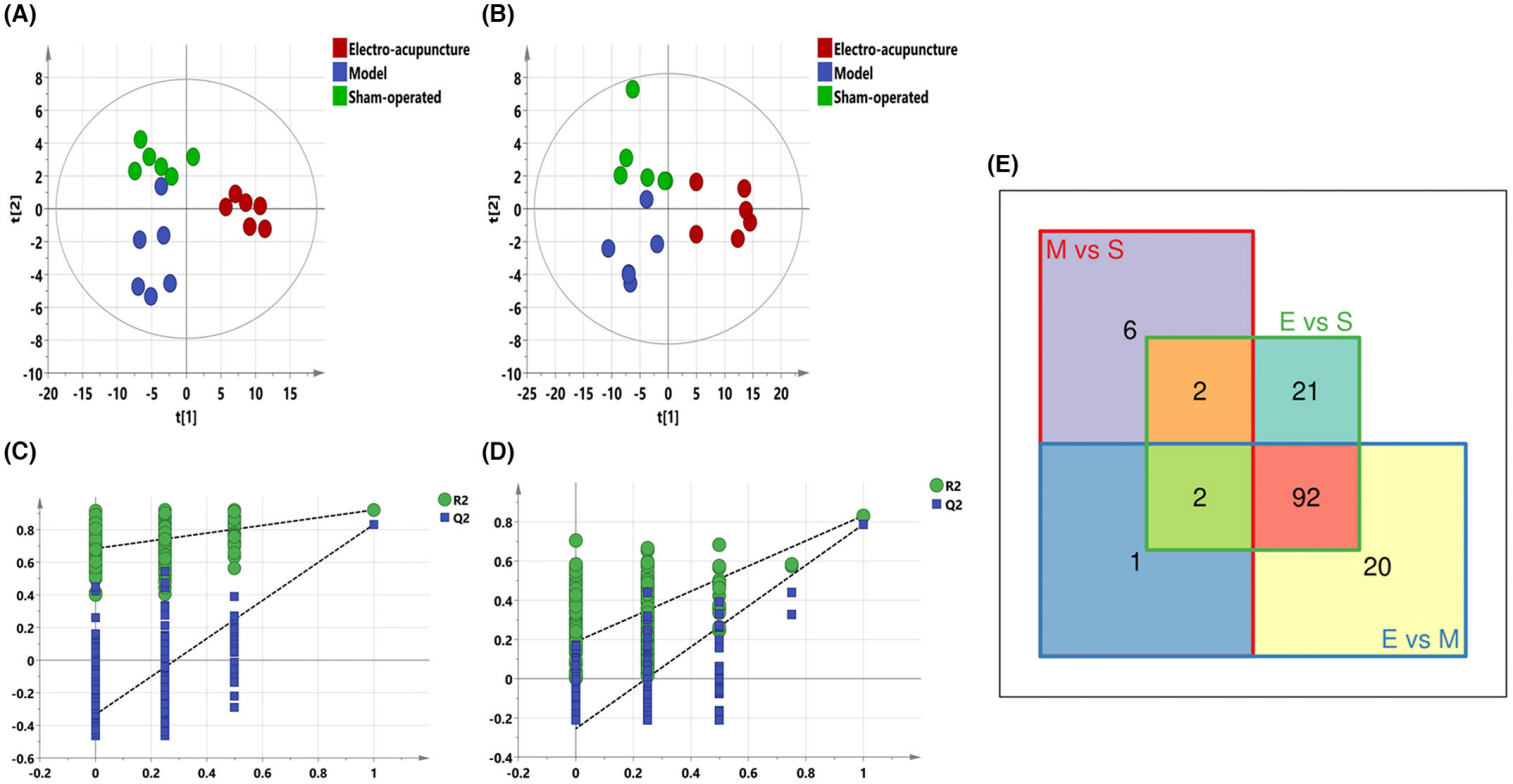
(A–D) PLS‐DA diagram of all samples. (A) PLS‐DA plot in negative ion mode (R^2^X 0.721, R^2^Y 0.963, Q^2^ 0.844), (B) PLS‐DA plot in positive ion mode (R^2^X 0.777, R^2^Y 0.907, Q^2^ 0.741), (C) Negative ion replacement test, (D) Positive ion replacement test. Figure 7E Venn diagram of the number of differential metabolites. The red box Model versus sham‐operated, the blue box electro‐acupuncture versus Model and the green box electro‐acupuncture versus sham‐operated.

PLS‐DA analysis was performed for all groups to observe the trend of the overall metabolic group of the three groups. The results are shown in Figure 6 (A–D), model, electro‐acupuncture and sham‐operated were distinguishable between all three groups, the model group was relatively close to the sham‐operated group and the electroacupuncture group was more clearly distinguished from the other two groups, suggesting the overall lipidome migrated more after electroacupuncture, which is generally consistent with previous results. All differential metabolites involved in the three groups were summarized by venn diagram (Figure 6E): the red box surrounded by the number of differential lipids for model versus sham‐operated, 11 in total; the blue box surrounded by the number of differential lipids for electro‐acupuncture versus model, 115 in total; and the green box surrounded by the number of differential lipids for electro‐acupuncture versus sham‐operated was 117. There are only two differential lipids common to all three groups. And 94 metabolites that overlap between electro‐acupuncture versus model and electro‐acupuncture versus sham‐operated (Table 4), mainly included glycerol esters, phospholipids and sphingolipids, and the trend of response changes after electroacupuncture was the same, which likewise indicated that the electroacupuncture had a much more pronounced effect on the serum lipid group than the model group.

**TABLE 4:** Common differential lipid information after electroacupuncture.

Name	Formula	Rt min	Fold change	
			E vs. M	E vs. S
TG 48:3	C_51_H_92_O_6_	14.94	0.29	0.27
TG 48:2	C_51_H_94_O_6_	15.42	0.31	0.28
TG 49:3	C_52_H_94_O_6_	15.22	0.34	0.32
TG 48:1	C_51_H_96_O_6_	16.00	0.38	0.34
TG 54:7	C_57_H_96_O_6_	14.62	0.33	0.34
TG 49:1	C_52_H_98_O_6_	16.26	0.38	0.34
TG 49:2	C_52_H_96_O_6_	15.72	0.37	0.35
TG 50:4	C_53_H_94_O_6_	14.96	0.37	0.36
TG 51:2	C_54_H_100_O_6_	16.24	0.42	0.38
TG 50:3	C_53_H_96_O_6_	15.47	0.41	0.39
TG 54:6	C_57_H_98_O_6_	15.04	0.38	0.39
TG 50:2	C_53_H_98_O_6_	15.97	0.45	0.42
TG 51:3	C_54_H_98_O_6_	15.71	0.46	0.42
TG 51:4	C_54_H_96_O_6_	15.26	0.44	0.43
TG 50:1	C_53_H_100_O_6_	16.57	0.49	0.45
TG 54:2	C_57_H_106_O_6_	17.12	0.49	0.47
TG 48:0	C_51_H_98_O_6_	16.62	0.48	0.47
TG 54:8	C_57_H_94_O_6_	14.59	0.44	0.48
TG 56:3	C_59_H_108_O_6_	17.15	0.53	0.51
LPC 16:1	C_24_H_48_NO_7_P	1.92	0.49	0.51
TG 52:1	C_55_H_104_O_6_	17.18	0.53	0.52
LPC 20:5	C_28_H_48_NO_7_P	1.62	0.55	0.52
DG 36:3	C_39_H_70_O_5_	11.73	0.59	0.52
TG 53:3	C_56_H_102_O_6_	16.32	0.56	0.52
TG 52:5	C_55_H_96_O_6_	15.09	0.51	0.52
TG 52:2	C_55_H_102_O_6_	16.56	0.57	0.53
LPC 20:3	C_28_H_52_NO_7_P	2.43	0.52	0.55
DG 36:4	C_39_H_68_O_5_	11.01	0.59	0.55
TG 52:5	C_55_H_96_O_6_	15.95	0.61	0.55
TG 56:6	C_59_H_102_O_6_	15.02	0.62	0.55
TG 53:4	C_56_H_100_O_6_	15.79	0.58	0.56
TG 54:3	C_57_H_104_O_6_	16.57	0.57	0.57
TG 54:5	C_57_H_100_O_6_	15.52	0.56	0.57
PE 36:2	C_41_H_78_NO_8_P	11.34	0.53	0.60
TG 54:4	C_57_H_102_O_6_	15.99	0.58	0.61
PC 32:1	C_40_H_78_NO_8_P	9.95	0.57	0.61
PE 34:2	C_39_H_74_NO_8_P	10.42	0.57	0.63
TG 56:4	C_59_H_106_O_6_	16.52	0.66	0.67
LPC 17:1	C_25_H_50_NO_7_P	2.33	0.65	0.68
PE 36:4	C_41_H_74_NO_8_P	10.22	0.60	0.69
LPC 19:1	C_27_H_54_NO_7_P	3.34	0.67	0.70
PC 38:4	C_46_H_84_NO_8_P	10.33	0.65	0.72
PC 36:5	C_44_H_78_NO_8_P	9.23	0.67	0.73
TG 52:3	C_55_H_100_O_6_	15.99	0.76	0.74
TG 52:4	C_55_H_98_O_6_	15.51	0.73	0.75
PC 40:7	C_48_H_82_NO_8_P	9.7	0.68	0.75
LPC 20:2	C_28_H_54_NO_7_P	3.03	0.75	0.76
PC 36:3	C_44_H_82_NO_8_P	10.36	0.73	0.78
TG 56:5	C_59_H_104_O_6_	16.26	0.78	0.78
PE 38:4	C_43_H_78_NO_8_P	11.16	0.70	0.79
PC 34:3	C_42_H_78_NO_8_P	9.17	0.74	0.79
PC 34:1	C_42_H_82_NO_8_P	10.89	0.83	0.81
TG 56:7	C_59_H_100_O_6_	15.59	0.69	0.82
LPC 18:2	C_26_H_50_NO_7_P	2.13	0.81	0.82
PC 38:5	C_46_H_82_NO_8_P	10.00	0.80	0.86
LPC 14:0	C_22_H_46_NO_7_P	1.78	0.90	0.86
PC 38:2	C_46_H_88_NO_8_P	11.84	0.81	0.87
PC 34:2	C_42_H_80_NO_8_P	10.13	0.93	0.91
PC 36:2	C_44_H_84_NO_8_P	11.09	1.04	1.04
PC 38:6	C_46_H_80_NO_8_P	9.63	1.11	1.09
PC 36:4	C_44_H_80_NO_8_P	9.94	1.08	1.09
PC 40:6	C_48_H_84_NO_8_P	10.64	1.16	1.12
LPC 18:0	C_26_H_54_NO_7_P	3.56	1.20	1.14
PC 38:4	C_46_H_84_NO_8_P	10.92	1.11	1.17
PC 34:0	C_42_H_84_NO_8_P	11.73	1.22	1.20
SM 42:1;O2	C_47_H_95_N_2_O_6_P	13.26	1.21	1.26
SM 43:2;O2	C_48_H_95_N_2_O_6_P	12.84	1.34	1.27
LPC 17:0	C_25_H_52_NO_7_P	3.2	1.29	1.27
SM 43:1;O2	C_48_H_97_N_2_O_6_P	13.46	1.28	1.33
Cer 41:1;O2	C_41_H_81_NO_3_	13.66	1.20	1.35
SM 33:1;O2	C_38_H_77_N_2_O_6_P	9.26	1.45	1.38
CE 20:4	C_47_H_76_O_2_	15.68	1.47	1.39
SM 40:1;O2	C_45_H_91_N_2_O_6_P	12.59	1.28	1.39
SM 40:2;O2	C_45_H_89_N_2_O_6_P	11.73	1.48	1.40
PC 36:0	C_44_H_88_NO_8_P	12.50	1.35	1.42
Cer 40:1;O2	C_40_H_79_NO_3_	13.33	1.35	1.42
SM 41:1;O2	C_46_H_93_N_2_O_6_P	12.92	1.30	1.44
PE O‐38:6	C_43_H_76_NO_7_P	10.72	1.30	1.45
PC O‐36:5	C_44_H_80_NO_7_P	10.38	1.37	1.53
HexCer 42:1;O2	C_48_H_93_NO_8_	13.42	1.56	1.55
CE 22:4	C_49_H_80_O_2_	16.14	1.53	1.57
PE O‐40:6	C_45_H_80_NO_7_P	11.57	1.50	1.59
PE O‐36:5	C_41_H_74_NO_7_P	10.67	1.39	1.60
SM 36:1;O2	C_41_H_83_N_2_O_6_P	10.88	1.69	1.63
PE O‐40:5	C_45_H_82_NO_7_P	12.37	1.53	1.66
PE O‐40:7	C_45_H_78_NO_7_P	11.28	1.55	1.67
PE O‐38:5	C_43_H_78_NO_7_P	11.58	1.47	1.68
CE 22:6	C_49_H_76_O_2_	15.35	1.92	1.72
PE O‐38:5	C_43_H_78_NO_7_P	11.33	1.62	1.76
CE 18:2	C_45_H_76_O_2_	15.98	1.86	1.80
PE O‐40:5	C_45_H_82_NO_7_P	12.15	1.58	1.81
CerPE 38:1;O2	C_40_H_81_N_2_O_6_P	10.40	2.00	2.00
PE O‐40:4	C_45_H_84_NO_7_P	12.26	1.73	2.02
PE O‐40:4	C_45_H_84_NO_7_P	12.47	1.79	2.05

### 3.3 Metabolomics pathway analysis of SD rat venous serum after electro‐acupuncture

**FIGURE 7:**
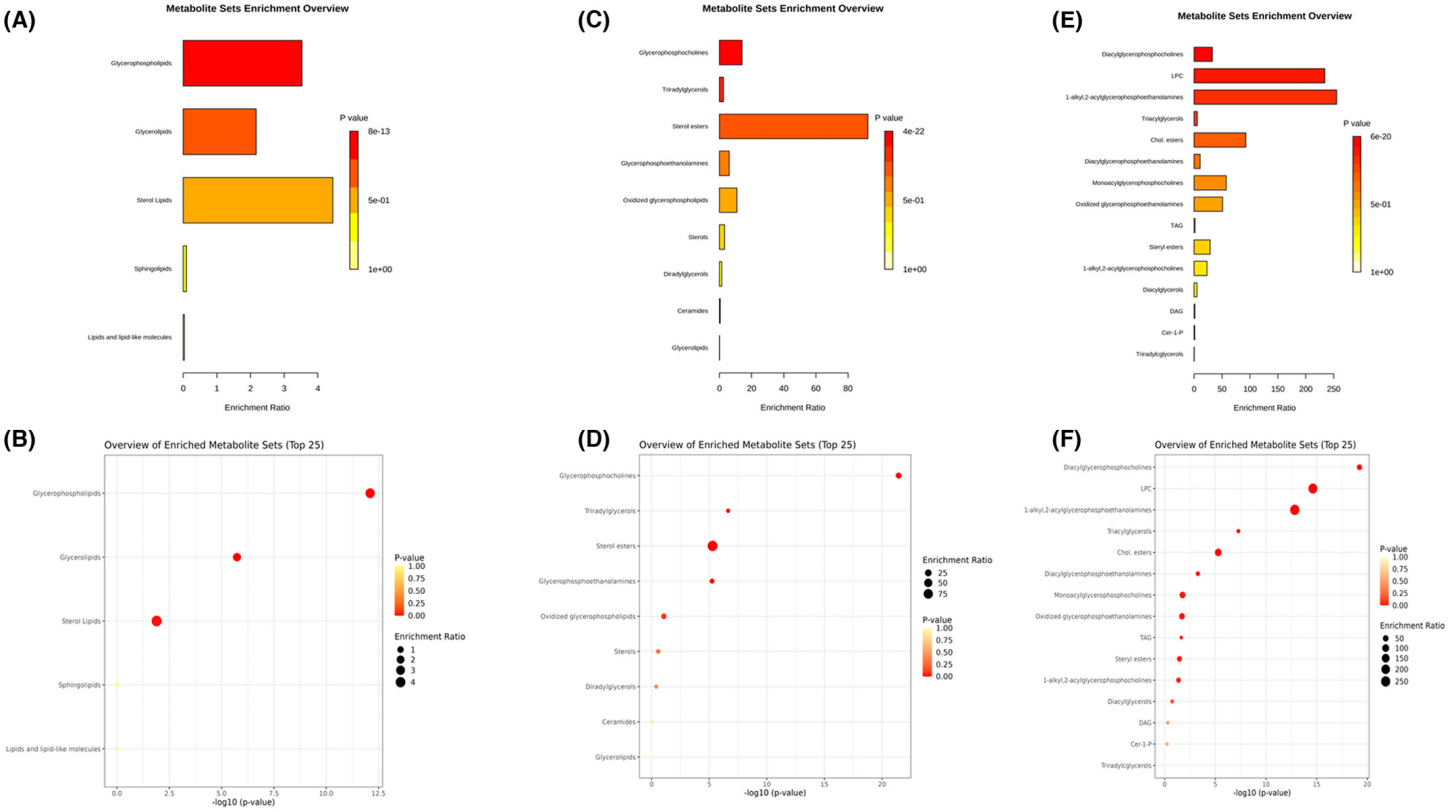
Lipid metabolism enrichment analysis. (A) Top lipid classification, enrichment analysis bar chart. (B) Top lipid classification, enrichment analysis dot plot. (C) Intermediate lipid classification, enrichment analysis bar chart. (D) Intermediate lipid classification, enrichment analysis dot plot. (E) The lowest lipid classification, enrichment analysis bar chart. (F) The lowest lipid classification, enrichment analysis dot plot.

The results of pathway enrichment analysis from different lipid classification levels showed that electroacupuncture mainly affected the lipids associated with four pathways: cholesteryl esters, sphingolipids, glycerides and phospholipids (the redder the colour, the more significant the expression of the pathway; the longer the bar or the larger the point, the more lipids matched in the pathway). And the metabolic pathways were further refined and analysed from the intermediate and lowermost lipid categories, see Figure 7 (A–F).

### 3.4 Post‐operative wound perfusion volume and area changes

**FIGURE 8:**
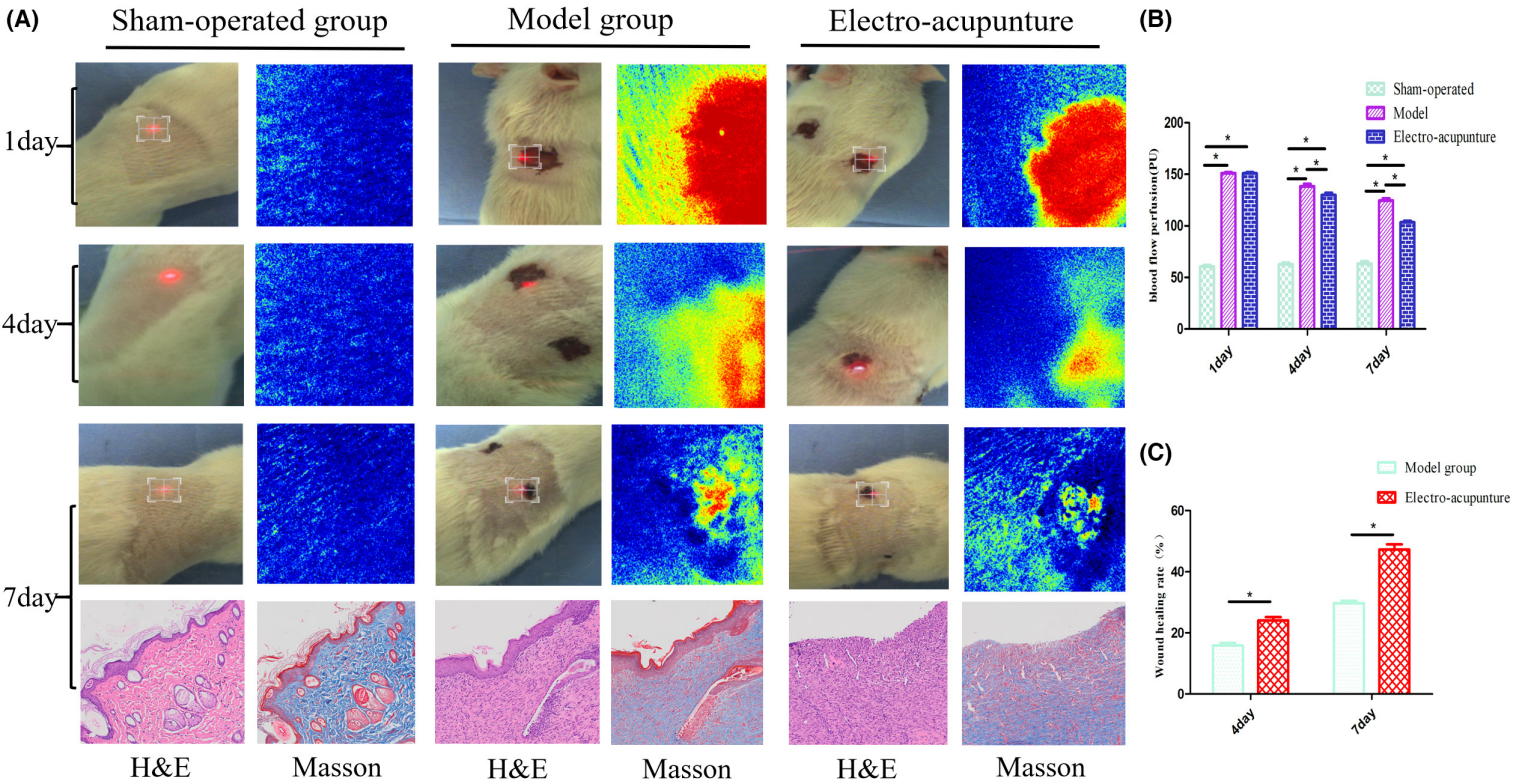
(A) The wounds of the three groups at different time points after surgery, the corresponding blood perfusion imaging images and the histological examination results of the three groups at 7 days after operation (X200) were compared. (B) Comparison of wound blood perfusion volume in the three groups at different time points after surgery. (C) Comparison of wound healing rate between Model group and electro‐acupunture at different time points after surgery.

On post‐operative day 1, the haemoperfusion volume in the model and electroacupuncture groups was comparable but significantly greater than that in the sham‐operated group, and the difference was statistically significant (*p* < 0.05). On post‐operative days 4 and 7, the perfusion volume of the model group and electroacupuncture group gradually decreased, but the perfusion volume of the model group was greater than that of the other two groups, and the difference was statistically significant (*p* < 0.05) and the results are shown in Figure 8 (A,B).On post‐operative days 4 and 7, the wound healing rate of the electroacupuncture group was faster than that of the model group, and the difference was statistically significant (*p* < 0.05), and the results are shown in Figure 8C.

### 3.5 Histological and immunohistochemical results

On the 7th post‐operative day, epithelial cells and fibroblasts were hyperplastic in the electro‐acupuncture group, collagen fibres were densely arranged, muscle fibres were more abundant and a thinner new epidermal layer was visible. And the skin repair status of the model group was weaker than that of the electroacupuncture group. See Figure 8A.

## 4 DISCUSSION

The stratum corneum (SC) of the skin plays a key role in the formation and maintenance of the skin barrier, and lipids are an important component of the SC, forming a well‐structured lipid matrix.^15^, ^16^ Differentiated keratin‐forming cells in the epidermis synthesize cholesterol, ceramide (CER), and free fatty acids (FFA), which form the major lipid classes present in the SC.^17^ Therefore, the metabolic processes of these lipids may play a key role in the skin wound repair process.

In this study, changes in lipid metabolism of serum were found in both the electroacupuncture and model groups. There were 11 differential metabolites for model versus sham‐operated, 115 differential metabolites for electro‐acupuncture versus model, and 117 differential metabolites for electro‐acupuncture versus sham‐operated. There are two differential lipids common to all three groups. And 94 metabolites that overlap between electro‐acupuncture versus model and electro‐acupuncture versus sham‐operated (Figure 4), mainly included glycerol esters, phospholipids and sphingolipids, and the trend of response changes after electroacupuncture was the same. Further metabolic pathway analysis revealed that electroacupuncture mainly affected lipids associated with four pathways: cholesteryl esters, sphingolipids, glycerol esters and phospholipids, which was consistent with the results of differential metabolite analysis. Yeom M et al^18^ have used electroacupuncture to treat hyperlipidaemia rats, and the results showed that it can significantly reduce the content of triglyceride and total cholesterol in serum and improve the level of serum lipid metabolism. In addition, the haemoperfusion volume and wound healing rate of the electroacupuncture group was faster than that of the model group. Histological and immunohistochemical also revealed that the electroacupuncture group had faster skin repair than the model group. These results suggest that electroacupuncture may promote skin wound repair in rats by mobilizing the lipid metabolism function of serum and improving local haemoperfusion volume. Therefore, our study may provide a safer and more effective complementary and alternative therapy for skin wound healing and provide an experimental basis for the study of the mechanism of electroacupuncture to promote skin wound healing.

Phospholipids not only constitute an important component of cell membranes, but also participate in the regulation of various cellular functions and play an important role in skin wound repair. Transcriptomic and lipidomic data revealed that Narciclasine extensively regulated lipid metabolism‐related genes, especially the Phospholipase A2 (PLA2) family, and increased anti‐inflammatory lipid molecules.^19^ Lysophospholipids and FFA (especially arachidonic acid) are the main products of phospholipids catalysed by PLA2.^20^ Among them, lysophosphatidylcholine (lysoPC) stimulates leukocyte activation, T‐lymphocyte chemotaxis and inflammatory cell accumulation.^21^, ^22^ Prostaglandins (PG) and leukotrienes (LT) produced by the PLA2‐mediated arachidonic acid cascade reaction can activate nuclear factor‐kB through the tumour necrosis factor signalling pathway, thus effectively promoting the division and activation of keratin‐forming cells.^23^ It has also been shown that exogenous phosphatidylcholine accelerates the wound healing process, allowing keratinocytes to complete proliferation and migration to form a new layer of keratin in a relatively short period of time.^24^ Oral administration of lactophospholipids to hairless mice exposed to UV‐B radiation‐induced photoaging resulted in downregulation of protein expression of nuclear factor kappa‐B (NF‐κB) and phosphorylated IκB‐α (κB‐α inhibitor), a significant decrease in the expression of pro‐inflammatory cytokines, especially tumour necrosis factor‐α, and improved skin hydration and barrier function.^25^ The skin barrier‐improving effect of lactoferrin appears to be associated with the activation of Nrf2‐keap1, which is associated with ROS scavenging. Lactoferrin activates Nrf2 and increases the expression of the antioxidant enzyme HO‐1, thereby reducing ROS produced by UV exposure.^26^ In addition, phosphatidylinositol also plays an important role in wound repair. Phosphatidylinositol is involved in the regulation of multiple signalling pathways in skin cells, including cell proliferation, apoptosis, and differentiation.^27^, ^28^ Li J et al^29^ have reported that electroacupuncture treatment can effectively decrease the over‐expression of related factors of phosphatidylinositol system in rats with acute cerebral infarction, improve cerebral autonomy movement, and alleviate cerebral vascular spasm. Our study revealed that phospholipid serum metabolites and metabolic pathways were mobilized by electroacupuncture intervention. It suggests that electroacupuncture may promote the balance of phospholipid metabolism by inhibiting excessive oxidative stress, accelerating SC recovery, regulating cell proliferation and migration, and suppressing inflammatory responses. This may be a metabolic mechanism of action of electroacupuncture to promote wound repair.

Sphingolipids are biologically active lipid molecules that are widely present in cell membranes and are involved in the regulation of a variety of life activities, being key components of skin barrier homeostasis and cellular processes such as apoptosis and stress response.^30^, ^31^ Impaired sphingolipid metabolism underlies several common skin pathologies, including atopic dermatitis, psoriasis and aging.^32^, ^33^ There are few studies on the effects of electroacupuncture on sphingolipid metabolism in skin repair. However, other studies have shown that electroacupuncture can regulate the changes of lipid metabolism in mice with post‐traumatic stress disorder, especially the content changes of sphingolipids, glycerides and fatty acyl groups.^34^ In the sphingomyelinase pathway, CER is formed by hydrolysis of sphingomyelins in the granular layer of the skin by sphingomyelinase. CERs are considered to be one of the most important epidermal sphingolipids, accounting for approximately 50% of the intercellular lipids of the SC^35^, ^36^ The CERs are transported from the endoplasmic reticulum to the Golgi apparatus, where the conversion to glucosyl CERs or sphingomyelin occurs.^37^ Signalling pathways such as protein kinase B (Akt), protein kinase C (PKC), mitogen‐activated protein kinase (MAPK), Jun amino‐terminal kinase (JNK), or phospholipase D (PLD) are regulated during CER stimulation.^38^ Although the importance of CER in skin cell proliferation and differentiation has long been known, in recent years sphingosine 1‐phosphate sphingosine (S1P) has also been found to be involved in processes such as the proliferation and differentiation of keratin‐forming cells. The ability of FTY720 to inhibit lymphocyte efflux was significantly diminished as shown by using a rat model with internalization‐resistant S1PR1.^39^ In S1PR1 knockout mice, enhanced macrophage migration is eliminated during inflammation regression.^40^ Incubation of macrophages with S1P appears to have an anti‐inflammatory effect, as the production of the pro‐inflammatory cytokines TNF‐α, IL‐6, IL‐12 and CCL2 is significantly reduced after activation by lipopolysaccharide (LPS).^41^ In addition, by increasing sphingolipid content and altering sphingolipid metabolic pathways, cell proliferation and differentiation can be promoted, which in turn promotes wound healing.^42^, ^43^ Dietary Sphingomyelin may improve skin barrier function by altering skin inflammation and covalently‐bound ω‐hydroxy CERs and may promote epidermal keratinized envelope formation by altering inflammation‐associated gene expression.^44^ Our study found that sphingolipids and their metabolic pathways migrated more after electroacupuncture intervention relative to the sham‐operated and model groups, suggesting that electroacupuncture may have promoted skin wound healing by promoting intra‐traumatic cell migration and regeneration, regulation of inflammatory response and immune response, enhancement of phagocytosis of immune cells, and improvement of the consequent skin barrier function. This may be another metabolic mechanism of action of electroacupuncture to promote skin wound healing.

**FIGURE 9:**
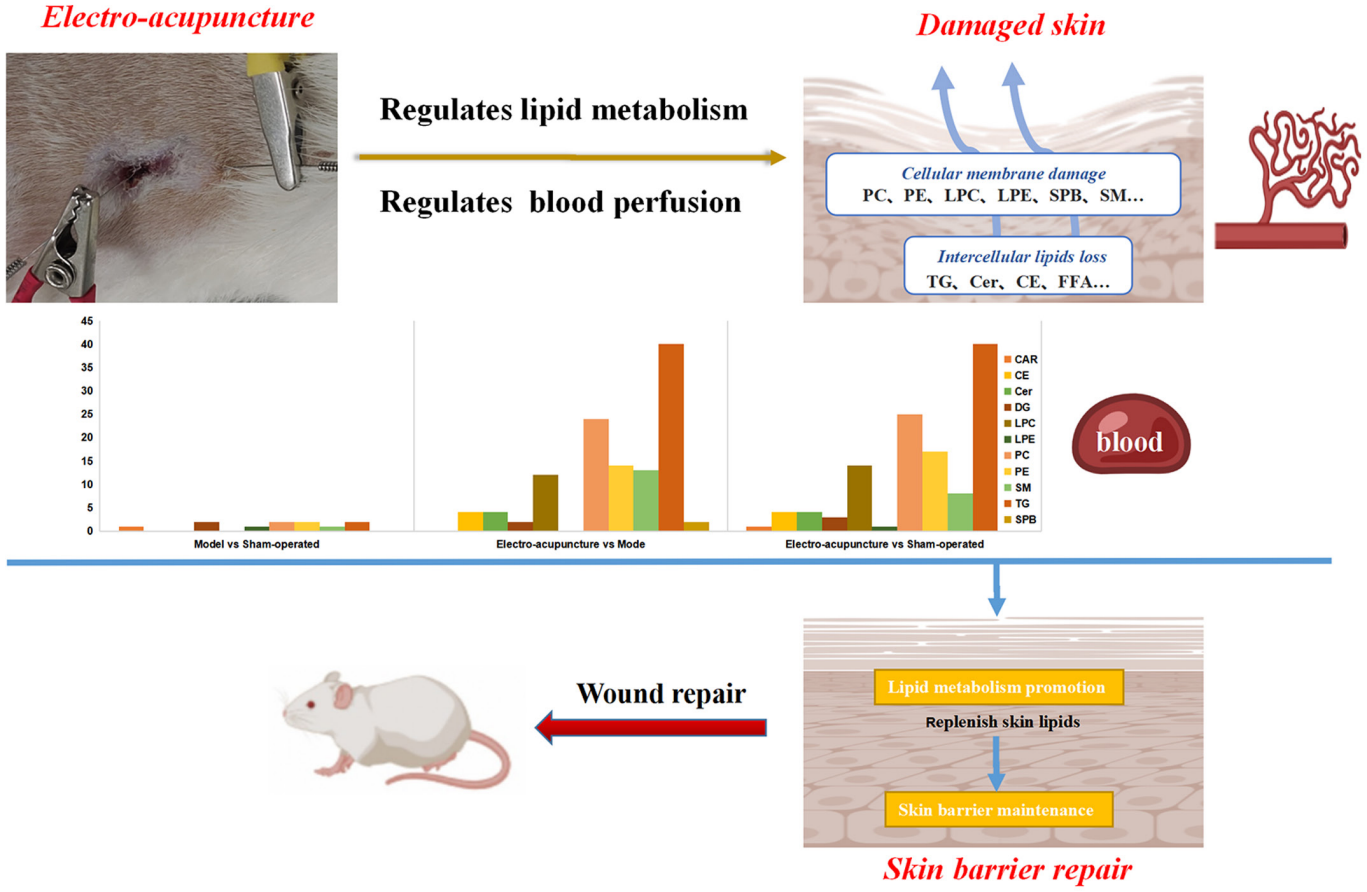
Schematic diagram of electroacupuncture promoting skin wound repair by regulating serum lipid metabolism and local blood perfusion.

In conclusion, as shown in Figure 9, our results suggest(that metabolites and metabolic pathways such as serum cholesteryl esters, sphingolipids, glycerides and phospholipids migrate more after electroacupuncture intervention relative to the sham and model groups and may play a key role in the mechanism of action of electroacupuncture to promote skin wound repair. In the process of skin wound healing in rats, electroacupuncture treatment helped restore the amount of wound perfusion, could promote the proliferation of epithelial cells and fibroblasts, attenuated the inflammatory response and lipid peroxidation in rat wounds, and improved serum lipid metabolism in rats. Although there are some limitations of our study, such as the urgent need for more experimental studies to verify the mechanism of action of electroacupuncture intervention on the regulation of serum lipid metabolism during skin wound repair, our results provide a new direction for the treatment of skin wound repair.

## AUTHOR CONTRIBUTIONS

**weibin du:** Funding acquisition (lead); project administration (equal); writing – original draft (lead); writing – review and editing (lead). **Lihong He:** Conceptualization (equal); writing – original draft (equal); writing – review and editing (equal). **Zhenwei Wang:** Conceptualization (equal); data curation (equal); writing – original draft (equal). **Yi Dong:** Conceptualization (equal); data curation (equal); formal analysis (equal). **Xiaofen He:** Formal analysis (equal); resources (equal); supervision (equal). **Jintao Hu:** Funding acquisition (equal); methodology (equal); supervision (equal). **Min Zhang:** Methodology (lead); project administration (lead); supervision (lead); writing – review and editing (lead).

## FUNDING INFORMATION

This work is supported by National Natural Science Foundation of China (NO. 81904053). Special Research Project of the Affiliated Hospital of Zhejiang Chinese Medical University (NO. 2021FSYYZY43). Hangzhou Medical and Health Technology Planning Project (NO. B20220021, B20200032, A20220507), Hangzhou Science and Technology Planning Project (NO. 2020ZDSJ0042, 20220919Y084). Zhejiang Province Traditional Chinese Medicine Science and Technology Project (NO. 2023ZR046, 2022ZB232), Research Project of Zhejiang Chinese Medical University (NO. 2021JKZKTS057B).

## CONFLICT OF INTEREST STATEMENT

All the authors declare that they have no conflicts of interest.

## CONSENT

Not applicable.

## Data Availability

All data generated and/or analyzed during this study are included in this published article.
